# Identification of Metabolic Pathway Systems

**DOI:** 10.3389/fgene.2016.00006

**Published:** 2016-02-10

**Authors:** Sepideh Dolatshahi, Eberhard O. Voit

**Affiliations:** Department of Biomedical Engineering, Georgia Institute of TechnologyAtlanta, GA, USA

**Keywords:** dynamic flux estimation (DFE), identifiability, metabolic pathway analysis, parameter estimation, pathway structure, underdetermined system of fluxes

## Abstract

The estimation of parameters in even moderately large biological systems is a significant challenge. This challenge is greatly exacerbated if the mathematical formats of appropriate process descriptions are unknown. To address this challenge, the method of dynamic flux estimation (DFE) was proposed for the analysis of metabolic time series data. Under ideal conditions, the first phase of DFE yields numerical representations of all fluxes within a metabolic pathway system, either as values at each time point or as plots against their substrates and modulators. However, this numerical result does not reveal the mathematical format of each flux. Thus, the second phase of DFE selects functional formats that are consistent with the numerical trends obtained from the first phase. While greatly facilitating metabolic data analysis, DFE is only directly applicable if the pathway system contains as many dependent variables as fluxes. Because most actual systems contain more fluxes than metabolite pools, this requirement is seldom satisfied. Auxiliary methods have been proposed to alleviate this issue, but they are not general. Here we propose strategies that extend DFE toward general, slightly underdetermined pathway systems.

## Introduction and background

A Google Scholar search for the keyword “parameter estimation” yields over 3 million hits, which renders it abundantly evident that the topic is everything but trivial, especially for applications in biology. The challenges of finding optimal parameter values for biological systems are multifold and include mathematical, statistical, computational, and even biological aspects. Mathematical issues include dependencies among parameter values, sloppiness, and different types of exact or approximate compensation between errors among the equations of the system, within equations, and even within terms of the equations. Computational challenges are driven by the sheer size of the often high-dimensional parameter space, the need to solve systems of differential equations thousands of times, and an error structure between model results and biological data that can be incredibly rough and contain uncounted local minima where search algorithms can get trapped. Biological issues include the size and complexity of a system, noisy or missing data, ill-characterized processes, and unrealistic parameter values. All these challenges are tightly interwoven and often create situations where no (good) solutions are obtained, where too many possible solutions can be identified, or where the exclusive criterion of the quality of the fit is misleading.

Partial help for overcoming some of these complications was provided by the insight that systems of ordinary differential equations (ODEs) can be estimated in a much simplified manner, at least to some degree. Namely, if data are available as time series measurements, and if it is possible to estimate the slopes of these time courses with some reliability, then the derivatives on the left-hand sides of the ODEs can be replaced with estimated slopes at many time points (Varah, 1982; Voit and Savageau, 1982a,b; Voit, 2000; Voit and Almeida, 2004; Chou and Voit, 2009; Jia et al., 2011). Consequently, each ODE, evaluated at a set of time points, is replaced with a purely algebraic system of equations, where the fluxes constitute its unknown variables. Each of these sets can be evaluated independently of all other sets and does no longer require numerical integration, which can account for more than 95% of the computational cost when parameters are directly estimated for ODEs (Voit and Almeida, 2004). The initial estimation of slopes from the time course data can be accomplished with a variety of methods that range from primitive to sophisticated (e.g., see Whittaker, 1923; Voit and Savageau, 1982b; Eilers, 2003; Voit and Almeida, 2004; Vilela, 2007, 2008; Dolatshahi et al., 2014 and discussions therein).

While it certainly simplifies parameter estimation, the slope estimation and decoupling method is not without its own issues. In particular, it may “warp” solutions in the direction of time, so that, for instance, oscillations have a predicted frequency that is too high or too low (see Chapter 5 of Voit, 2012). Nonetheless, the method can serve as an effective first stab at a complicated problem and thereby provide reasonable initial guesses for standard estimation techniques.

A prerequisite for any parameter estimation effort is knowledge of the mathematical formats of all involved processes, or at least a set of reasonable assumptions regarding these formats, because they obviously dictate the role of each parameter. However, guidelines regarding optimal formats for biological process descriptions are not provided by nature. Linear functions have been very successful in engineering, but it has become clear that they are inadequate for representing many biological phenomena. Thus, one needs to resort to non-linear representations, of which, of course, there are infinitely many. One could argue that biological systems must satisfy the laws of physics, but it is usually impossible to deconvolve biological processes neatly into physical components that can be represented based on physical theory (Voit et al., 2010; Voit, 2013a). To circumvent this problem, many biological systems modelers tend to use certain default representations that have a justification in specific, and often simplified instances but do certainly not tell the whole truth about a biological system *in vivo* or are valid in other contexts (Voit et al., 2015). Arguably the best studied example is the Michaelis–Menten rate law, which is approximately true in carefully crafted experiments *in vitro*, but whose prerequisites are most certainly violated in actual biological systems *in situ* (Savageau, 1992, 1995). Similarly, mass action functions in biochemistry, SIR models in epidemiology, and Lotka–Volterra models in ecology may be excellent starting points for the design of models, but it is quite evident that they cannot truly capture the full complexity of living systems in all its details.

One might think that it does not matter too much if the functional form is not perfect, as long as all data of interest are fit with sufficient accuracy. This argument may be true if future predictions and explanations only pertain to the data ranges used for model parameterization. However, as soon as the model is extrapolated into new ranges of its state variables, extrapolations with the wrong model may lead to grossly unsatisfactory results (Goel et al., 2008). One root cause of such extrapolation problems is a compensation of errors, which may occur within fluxes, among fluxes of the same equation, and among fluxes of different equations. While such compensation can lead to acceptable residual errors in the original data fit, extrapolations to new conditions can become rather unreliable; for specific details see Supplements of Goel et al. (2008).

Faced with this conundrum, the method of dynamic flux estimation (DFE) was suggested for the analysis of metabolic time series data (Goel et al., 2008). In principle, DFE could be applicable to any types of ODE systems, such as gene regulatory networks that offer similar identification challenges (Siegenthaler and Gunawan, 2014; Ud-Dean and Gunawan, 2014), but a very beneficial feature of metabolic systems is the conservation of mass at each metabolite pool, which has as a consequence that many fluxes appear in more than one equation. It will become evident throughout this article that this fact is important for DFE.

**Figure 1:**
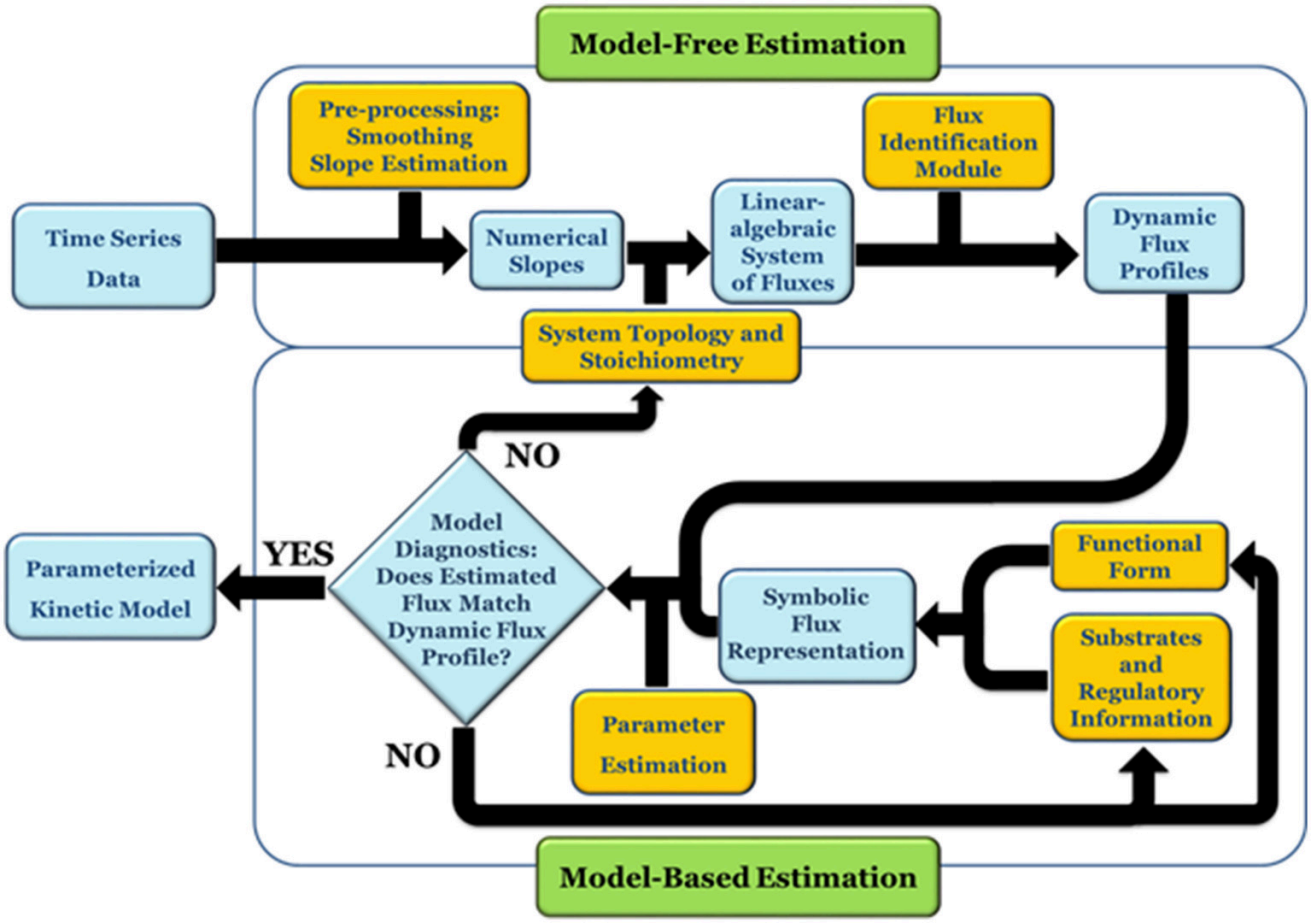
**DFE is a model characterization strategy and consists of two phases (adapted from Goel et al., 2008)**. In the first, model-free estimation phase, it takes time series of concentration data as input and estimates the dynamic flux profiles, which in turn are used as input to phase 2, which consists of a model-based estimation. In this phase, functional forms and regulatory assumptions are incorporated and parameters are estimated for each flux separately.

DFE consists of two phases, the first of which is model-free and makes very few assumptions (Figure 1). It includes data preprocessing, time course smoothing, the estimation of slopes of the smoothed time courses, and the solution of linear algebraic systems. Generically, each equation of the ODE is written as

(1) $$\begin{array}{c} \begin{array}{l} \frac{dX_{i}}{dt}=Influx_{1}^{i}+Influx_{2}^{i}+Influx_{3}^{i}+\ldots \\ \;-Efflux_{1}^{i}-Efflux_{2}^{i}-\ldots \end{array} \end{array}$$

**Figure 2:**
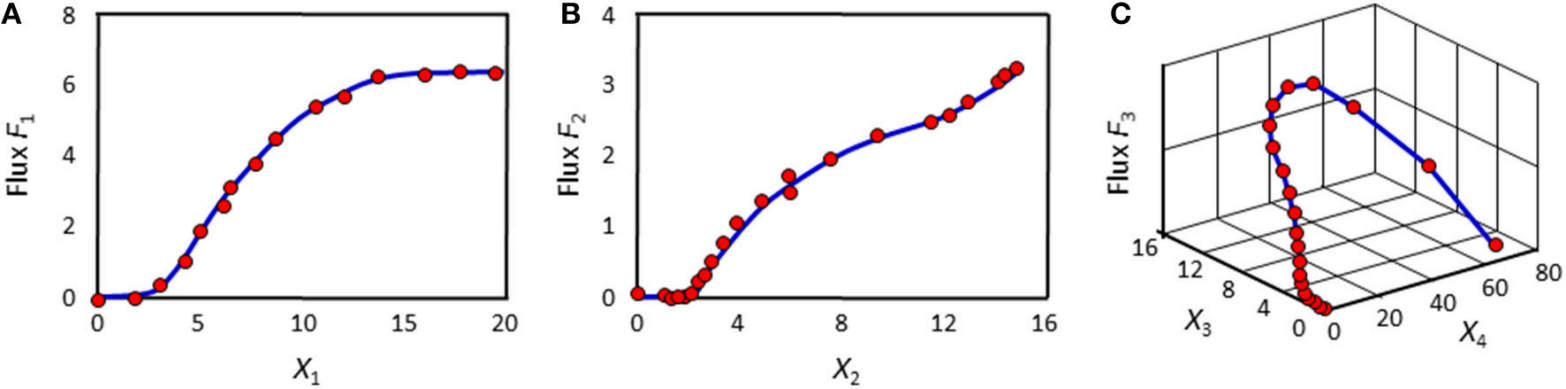
**Example results of the first phase of DFE**. The flux in panel **(A)** might be representable with a Hill or logistic function. The fluxes in panels **(B)** and **(C)** are adapted from Chou and Voit (2012); their optimal mathematical formats are unknown.

At each time point, the left-hand side is replaced by the appropriate slope, and the equations are simultaneously valid for all time points. The ultimate result of this phase consists of numerical or graphical time series profiles of all fluxes; in other words, the analysis yields plots of the fluxes in the system against time or against metabolites and modulators. Importantly, this phase does not reveal functional formats (Figure 2).

The second phase of DFE is dedicated to the mathematical characterization and parameterization of each flux profile. This phase requires the assumption of functional formats, which are fitted against the numerical flux representations. This step requires parameter estimation, but it is much simpler than the estimation of the original ODE systems, because it now targets explicit functions of one or a few variables in isolation and with correspondingly few parameters. For instance, the graphical result in Figure 2A might suggest a Hill or logistic function as a reasonable format, while appropriate formats for the trends in Figures 2B,C are not clear. It should be noted that this estimation of individual fluxes avoids many of the error compensation issues mentioned before.

Once a mathematical format is chosen for a particular flux, the data are fitted against this alleged format or against a roster of candidate functional forms. No generic strategies exist at this point for selecting candidates or proving their optimality, and it might be useful to scan through a list of candidate functions; for a similar approach in statistics, see Sorribas et al. (2000). Within this list, one may then attempt to identify the best fitting format through regression diagnostics, such as the residual error and a *run test for residuals* (Draper and Smith, 1981). The special case of the power-law format simplifies this step (Savageau and Voit, 1982), as a logarithmic transformation yields linearity and thus permits testing of the appropriateness of a functional form with diagnostic methods of multiple linear regression, even though one has to consider the distortion of the error structure due to the transformation. It is possible that several candidate functions are equally plausible and lead to similar fits. For instance, a Hill function and a logistic function can have essentially indistinguishable graphs. It is also possible that no functional form may be capable of yielding a reasonable fit, which may suggest the existence of missing features in the models, such as regulatory signals that had not been taken into account in the assumed pathway structure. Such suggestions correspond to novel hypotheses that are testable with further experiments and may lead to biological discoveries, as was demonstrated in Dolatshahi et al. (2016a).

The first phase of DFE mandates that an algebraic system of fluxes be solved at each time point (see Equation 1). This process is straightforward if the number of independent fluxes equals the number of dependent variables for which data exist. However, if the stoichiometric matrix of the system is not full-rank, which actually is the most common case, a direct inversion is not possible, and one needs to resort to auxiliary methods or mathematical operations that cast the problem in a simpler form (Jia et al., 2012; Liu and Gunawan, 2014). Unfortunately, such methods often necessitate additional biological information to make the stoichiometric matrix invertible (e.g., Voit, 2009; Chou and Voit, 2012; Iwata et al., 2013). As a consequence, these methods are seldom general and often require specific features of the data.

As an alternative or complementation of these methods, this article describes a generic flux identification procedure for slightly underdetermined systems and characterizes the space of available fluxes. The article furthermore discusses conceptual strategies for dealing with missing data and proposes mixed parameter estimation strategies when DFE is only partially applicable. This section involves the second, model-based phase of DFE.

In reality, biological data are always noisy and often incomplete, which adds uncertainty to any estimation or identification method. Indeed, noise, missing data, and estimation issues lead to a complicated intermixing of errors that are difficult to deconvolve. In order to focus exclusively on issues directly associated with the identification of fluxes, we decided here to use “ideal” data, which we generated with a published model (Curien et al., 2009). Many authors have discussed means of addressing and smoothing noisy data and dealing with less than ideal data (e.g., Vilela, 2007; Voit, 2011; Dolatshahi et al., 2014; and references therein), so that we will not revisit this issue here. However, we note that methods very similar to those presented here were recently applied to an actual, rather complex system (Dolatshahi et al., 2016a,b).

## Characterization of metabolic fluxes from time series data

If a pathway system is underdetermined, DFE cannot directly be applied. The issue in this case is not the absence of a solution; rather, the challenge is the existence of an entire space of feasible solutions and the need to decide which of these solutions are in some sense “better” than others. One could explore whether certain normalization or regularization procedures might help, but it appears that they do not solve the problem here, as we simply do not know what type of flux distribution nature considers optimal. For instance, the use of the Moore-Penrose pseudo-inverse (Penrose, 1955; Albert, 1972) yields a solution, but some fluxes of this solution are typically negative, which is often biologically unrealistic. Characterizability analysis (Voit, 2013b) reveals which fluxes within an underdetermined system can be estimated with DFE without additional information, but does not suggest further steps toward an optimal solution. The strategy of the following sections will be to study the entire set of feasible solutions in a drastically reduced space, whose dimension equals the number of the degrees of freedom within the stoichiometric system.

Along with the exploration of the solution space, useful strategies will be introduced to visualize feasible candidate sets. Initially, no information about the functional forms and the contributing metabolites and modulators of each flux is assumed to be available. Later on, minimal generic features of metabolic fluxes are suggested as constraints to improve the results. It is noted, though, that, even with these constraints, the solutions are not necessarily unique. Finally, solutions in the form of point-wise numerically defined fluxes will be suggested that are appropriate, if not optimal, according to certain criteria of biological reasonableness.

The source code for the following analyses has been deposited on github (https://github.com/sepidd/Identification-of-Metabolic-Pathway-Systems) and is also presented in the Supplementary Material.

### Mathematical formulation of the problem

A metabolic pathway system as formulated in Equation (1) can be written in general matrix and vector notation as

(2) $$\begin{array}{c} \frac{dX}{dt}=\dot{X}=Av. \end{array}$$

Here, ***X*** denotes a vector of *n* metabolite concentrations and ***v*** is a vector of *m* fluxes, i.e., reaction rates, while *A* is the stoichiometric matrix. The vectors, but not the matrix, change with time, and the functional forms governing the fluxes are functions of their substrates and regulators. They are in general unknown or based on assumptions that might or might not hold under the given experimental conditions or *in vivo*. Moreover, in certain cases, regulators and cofactors are yet to be discovered and are therefore falsely omitted. This uncertainty is the reason to attempt minimizing assumptions while executing the task of inferring flux profiles from metabolic time series data. At the same time, DFE provides us in this phase with the option of testing and challenging some of the prior assumptions and possibly discovering missing regulatory effects (cf. Dolatshahi et al., 2016a).

Assuming that data smoothing and slope estimation had been conducted successfully at each time point *t*_*i*_, we replace the left-hand side of Equation (2) with the vector of slopes at time *t*_*i*_, which we call *b*(*t*_*i*_). Equation (2) can thus be written as a set of algebraic equations. Specifically, suppose that ***b***(*t*) = ${\left[{\dot{X}}_{1}(t),\;\ldots ,\;{\dot{X}}_{n}(t)\right]}^{T}$ is the vector of slopes of dependent variables at time *t* and *A* is the *n* × *m* stoichiometric matrix, which is constant throughout the time period of any given experiment. Then we obtain directly the linear algebraic system

(3) $$\begin{array}{c} Av\left(t\right)=b\left(t\right) \end{array}$$

At a steady state, or when the numerical values of the derivatives are known, Equation (3) has a solution that can be computed for every time point by matrix inversion, if the system has full rank. However, most metabolic systems are under-determined, so that a unique solution does not exist.

We can thus distinguish three situations. (1) When the system has maximal rank, the solution is obtained with the regular inverse, so that $v\left(t_{i}\right)=\;A^{-1}\;b\left(t_{i}\right)$ is the solution of the system of equations. (2) When the system is over-determined and has more equations than unknowns (*m* < *n*), the Moore-Penrose pseudo-inverse *A*^+^of matrix *A* minimizes the sum of squared errors, $arg\;min\left(∥Av\left(t_{i}\right)-b\left(t_{i}\right)∥\right)\;=\;A^{+}\;b\left(t_{i}\right)$. This solution is equivalent to the result of linear regression. Finally, (3), the case of under-determined systems (*m* > *n*) is the most common situation in metabolic modeling, because most pathway systems contain more reaction steps than metabolites. This common occurrence makes the under-determined case particularly important for the model-free phase of DFE and suggests that we investigate if the pseudo-inverse solution $v\left(t_{i}\right)=A^{+}\;b\left(t_{i}\right)$ constitutes a biologically feasible, or even optimal, solution.

Pseudo-inverses have been used to solve under-determined systems for a long time. They are characterized by the minimum *L*^2^-norm within a one- or higher-dimensional space of admissible solutions, i.e., *arg min*(||*A**v***(*t*_*i*_) − ***b***(*t*_*i*_)||). While the best solution, in terms of the smallest norm, is guaranteed by the pseudo-inverse, the resulting fluxes are not necessarily positive, and there is no guarantee that they are biologically meaningful, let alone optimal. In fact, experience shows that minimum-norm solutions often include negative values, which are not biologically feasible as flux values, unless one permits flux inversion, which is not always realistic. The issue of under-determined systems in DFE has been known since the inception of the method, and characterizability analysis, based on pseudo-inverses, was introduced as an *a priori*, data-independent check for the applicability of DFE given a particular pathway system (Voit, 2013b).

### A compact representation: gamma-space and gamma-trajectory

In order to characterize the space of admissible flux sets $v\left(t\right)={\left[v_{1}\left(t\right),\;\ldots ,\;v_{m}\left(t\right)\right]}^{T}\;t\in \left[0,\infty \right)$ in an efficient manner, a more compact representation is desirable. For pathways with *m* fluxes and *n* dependent variables, where *m* > *n*, let *d* be the number of degrees of freedom (DOF): *d* ≥ *m*−*n*. Without loss of generality, we assume that the rank of the system is *n*. At each time point t, the space of solutions satisfying Equation (3) can be written as:

(4) $$\begin{array}{c} \begin{array}{l} v\left(t\right)=A^{+}b\left(t\right)+\left(I_{m}-A^{+}A\right)w(t)=A^{+}b\left(t\right)\\ \;+null\left(A\right)\gamma (t) \end{array} \end{array}$$

Here, *A*^+^ = *A*^*T*^(*AA*^*T*^)^−1^ is the Moore-Penrose pseudo-inverse, *A*^+^***b***(*t*) is the minimum-norm flux set at time *t*, and 𝕀_*m*_ is the *m* × *m* identity matrix. While *A*^+^***b***(*t*) is easily computed for practical applications with software like MATLAB, the result often contains one or more negative fluxes for some time points, which is usually undesirable. However, if ***w***(*t*_*i*_) is a vector of *m* arbitrary, real-valued elements, then the complete solution $v\left(t_{i}\right)=A^{+}b\left(t_{i}\right)+\left({𝕀}_{m}-A^{+}A\right)w\left(t_{i}\right)$ represents all possible solutions and spans the null space of the stoichiometric matrix *A*. In numerical evaluations, this null space is readily determined with the *null*(*A*) command in MATLAB.

The columns of *null*(*A*) = [*vec*_1_, *vec*_2_, ⋯, *vec*_*d*_] span the null space of *A*, and **γ**(*t*) = [γ_1_(*t*), γ_2_(*t*), ⋯ γ_*d*_(*t*)]^*T*^ is the corresponding vector of coefficients at time *t*. Each feasible solution of Equation (3) at time *t* can thus be uniquely represented by **γ**(*t*). This representation allows us to explore the *d*-dimensional *Gamma-space* instead of the feasible subset of the *m*-dimensional space of fluxes, whose visual representation is much more challenging.

The representations for all time points are now collected as follows. For each time point *t*, a feasible flux set ***v***(*t*) can be calculated by finding Gamma coefficients that satisfy ***v***_***n**ull*_ (*t*) = *null* (*A*) **γ**(*t*) = [*v*_1_ (*t*), …, *v_m_*(*t*)]^*T*^ − *A*^+^***b*** (*t*). This equation can be assessed by projecting ***v***_***n**ull*_(*t*) onto the vectors *vec*_1_, *vec*_2_, …, *vec*_*d*_, which span the null space of *A*. The coefficient vector [γ_1_(*t*), …, γ_*d*_(*t*)] constitutes a point in the *d*-dimensional Gamma-space, representing time point *t*, and the collection of these points constitutes a trajectory, which we call the *Gamma-trajectory*. Each Gamma-trajectory uniquely represents a feasible flux set traversing all time points, as long as this trajectory corresponds exclusively to non-negative fluxes.

**Figure 3:**
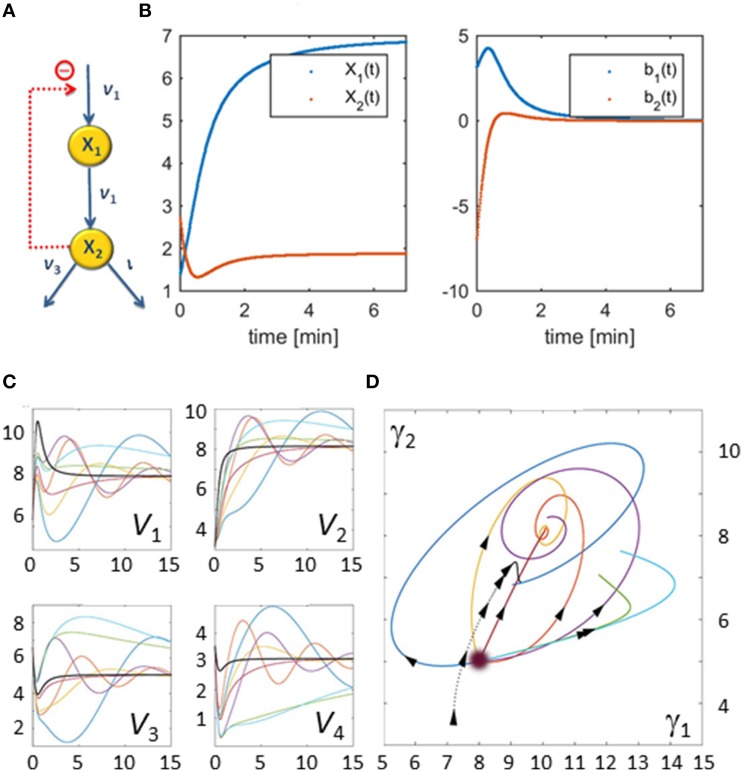
**Illustration example used to demonstrate the core concepts of the flux characterization procedure**. The pathway has a very simple structure as depicted in Panel **(A)**. Panel **(B)** shows **X**_1_(**t**) and **X**_2_(**t**) on the left and the slopes of **X**_1_(**t**) and **X**_2_(**t**) estimated from noise-free measurements on the right. Panel **(C)** shows seven examples of flux sets vs. time that satisfy Equation (5) exactly; for this illustration, all start at the same point as the original flux set (*v*(0) = [6.3271, 3.1588, 6.5486, 3.5486] corresponding to **γ**(0)^*T*^ = [8, 5]). The thicker black curves are the fluxes with which the original data were produced. The corresponding Gamma-trajectories are depicted with the same color scheme in Panel **(D)**. The blurry dot indicates the common start value of these trajectories while the dotted line represents the true flux, which is known in this artificial example.

As an illustration, let us consider a simple network consisting of two dependent variables and four fluxes (Figure 3A). Its stoichiometric representation is

(5) $$\begin{array}{c} \left[\begin{array}{cc} \begin{array}{cc} 1 & -1 \end{array} & \begin{array}{cc} 0 & 0 \end{array}\\ \begin{array}{cc} 0 & 1 \end{array} & \begin{array}{cc} -1 & -1 \end{array} \end{array}\right]\;\left[\begin{array}{c} \begin{array}{c} v_{1}\left(t\right)\\ v_{2}\left(t\right) \end{array}\\ \begin{array}{c} v_{3}\left(t\right)\\ v_{4}\left(t\right) \end{array} \end{array}\right]=\left[\begin{array}{c} b_{1}\left(t\right)\\ b_{2}\left(t\right) \end{array}\right] \end{array}$$

Suppose that metabolite concentrations *X*_1_(*t*) and *X*_2_(*t*) have been measured every 30 s between 0 and 15 min. Finding the slopes of the concentration trends directly yields *b*_1_(*t*) and *b*_2_(*t*) (Figure 3B). The feasible space of solutions, in terms of fluxes, is a two-dimensional plane within a 4-dimensional space that is difficult to visualize directly. Figure 3C shows some representative flux solutions. Even though these are very different, and several of them in fact have little similarity to the fluxes in the model used to generate the “data” (black curves in Figure 3C), all these fluxes satisfy Equation (5) exactly. The corresponding Gamma-trajectories are depicted in Figure 3D. The fluxes and Gamma-trajectory with which the concentration data were originally generated are shown in black in Figures 3C,D.

The solutions shown in Figure 3 are among the infinitely many admissible solutions generated by the following procedure, which actually only yields a small subset of all possible solutions. Starting at some initial point in the Gamma-space, a phase-plane trajectory is computed according to a stable linear state-space model of the form $\dot{\gamma }\left(t\right)=B\gamma \left(t\right)$. This is certainly not the only strategy for creating flux sets, but it constitutes a simple option that leads to continuous fluxes. A Monte-Carlo approach is utilized, in which a 2 × 2 matrix *B* is randomly generated, but where only those matrices *B* are retained that have negative real eigen values and result in non-negative fluxes for all time points. The resulting set of trajectories yields many dynamical fluxes with quite different features. Figure 3C shows some feasible solutions for fluxes *v*_1_ through *v*_4_ in multiple colors as thin lines, superimposed on the flux of the actual model, from which the concentration data were generated (black line). These fluxes are shifted in Panel **(C)**, so that their initial values match, in order to facilitate easier comparisons. Interestingly, the resulting fluxes can possess behaviors ranging from simple shoulder curves to over- and undershoots and different oscillatory responses. One notes that this Monte-Carlo strategy does not address issues of noise in the data, but is simply a means of retrieving diverse solutions that are mathematically admissible.

### Admissible subset of gamma-space: the subspace of non-negative fluxes

For biological realism, it is necessary to determine the set of **γ**'s for which the corresponding vector *v*(*t*) consists of non-negative values for all fluxes and all time points. According to Equation (4), the feasible space, given by ***v***(*t*) = *A*^+^***b***(*t*) + *null* (*A*) **γ** > **0**, is an intersection of *m* half-spaces:

(6) $$\begin{array}{c} A^{+}\left(i,:\right)b\left(t\right)+\gamma_{1}vec_{1,i}+\ldots +\gamma_{d}vec_{d,i}\geq 0\;i=1,\;2,\cdots ,m \end{array}$$

Here, *A*^+^(*i*, :) denotes the *i*^*th*^ row of the *m* × *n* Moore-Penrose pseudo-inverse matrix. The inequalities are linear and thus constitute a bounded or unbounded polytope.

### Formulating the problem as an optimization task

According to Equation (6), the solution set is still infinite, thus raising the question of whether biological constraints could be evoked to reduce the feasible space of solutions. A possibly pertinent constraint for the selection of meaningful flux profiles is the overall minimization of the magnitudes of positive fluxes, which might be interpreted as a form of metabolic energy conservation. Minimizing the sum of fluxes at steady state has been referred to as the *parsimonious enzyme effect* (Lewis, 2010). Here, the terminology is slightly different, as the minimization is done for the sum of all fluxes over all time points. Since the non-negativity constraints are already in place, this sum of fluxes at all time points equals the so-called “minimum *L*_1_-” or “Manhattan-” norm, which is defined as $\underset{\begin{array}{c} v\geq 0\\ Av=b \end{array}}{\min}{\left\|v\right\|}_{1}=\underset{\begin{array}{c} v\geq 0\\ Av=b \end{array}}{\min}\sum_{i\;=\;1}^{m}\left|v_{i}\right|=\underset{\begin{array}{c} v\geq 0\\ Av=b \end{array}}{\min}\sum_{i\;=\;1}^{m}v_{i}$. The optimization problem leading to this result in terms of γ is shown in Equation (7). The constraint *A**v*** = *b* is already taken into account, since the representation in Equation (4) only allows for fluxes that satisfy this constraint. Thus, the optimization simplifies to:

(7) $$\begin{array}{c} \begin{array}{l} \underset{A^{+}b\left(t\right)+null\left(A\right)\gamma (t)\geq 0}{\min}\sum_{i\;=\;1}^{m}A^{+}b\left(t\right)+null\left(A\right)\gamma (t)\\ \;=\underset{A^{+}b\left(t\right)+null\left(A\right)\gamma (t)\geq 0}{\min}\sum_{i\;=\;1}^{m}null\left(A\right)\gamma (t) \end{array} \end{array}$$

The important insight from Equation (7) is that the optimization problem can be translated into a simpler linear program in terms of **γ**(t), which can be solved using algorithms for linear programming, such as the simplex method. In practice, testing the corner points of the feasible polyhedron for identifying the corner with the minimum sum is a very well-established way of arriving at the optimal solution (Dantzig, 1984).

One should note that DFE and the choice of an objective function for the identification of biologically reasonable flux solutions are entirely independent, For instance, as an alternative optimization approach to minimizing the sum of fluxes for all time points, we could select the *L*_2_-norm of the flux vector at each point in time. This choice emphasizes and weighs the roles of the individual fluxes in a different manner. Minimizing the squared sum of fluxes at steady state has been referred to as *flux optimization* (Holzhütter, 2004). Again, our terminology is slightly different because the minimization pertains to all fluxes and all time points. This task is described in Equation (8) and again represents in some sense the minimum-energy flux set.

(8) $$\begin{array}{c} \underset{\begin{array}{c} v\geq 0\\ Av=b \end{array}}{\min}{\left\|v\right\|}_{2}{}^{2} \end{array}$$

The optimization problem in Equation (8) can be reformulated as the optimization problem of minimizing the *L*_2_-norm of the vector **γ**(*t*). Equation (9) shows this reformulation:

(9) $$\begin{array}{l} \underset{A^{+}b\left(t\right)+null\left(A\right)\gamma (t)\geq 0}{\min}{\left(A^{+}b\left(t\right)+null\left(A\right)\gamma \left(t\right)\right)}^{T}(A^{+}b\left(t\right)\\ +null\left(A\right)\gamma \left(t\right))=\underset{{}_{A^{+}b\left(t\right)+null\left(A\right)\gamma (t)\geq 0}}{\min}{\left(A^{+}b\left(t\right)\right)}^{T}A^{+}b\left(t\right)\\ +\gamma {\left(t\right)}^{T}null{\left(A\right)}^{T}null\left(A\right)\gamma \left(t\right)+\gamma {\left(t\right)}^{T}null{\left(A\right)}^{T}A^{+}b\left(t\right)\\ +{\left(A^{+}b\left(t\right)\right)}^{T}null\left(A\right)\gamma \left(t\right)=\underset{A^{+}b\left(t\right)+null\left(A\right)\gamma (t)\geq 0}{\min}\gamma {\left(t\right)}^{T}{\text{I}}_{m}\gamma \left(t\right)\\ =\underset{A^{+}b\left(t\right)+null\left(A\right)\gamma (t)\geq 0}{\min}{\left\|\gamma \left(t\right)\right\|}^{2} \end{array}$$

Here, *null* (*A*)^*T*^ *null* (*A*) = 𝕀_*m*_ is the identity matrix of dimension *m*, because the columns of *null*(*A*) are orthonormal base vectors of the null space. Furthermore, the pseudo-inverse solution *A*^+^***b***(*t*) is orthogonal to the null space, so that *null*(*A*)^*T*^*A*^+^***b***(*t*) = (*A*^+^***b***(*t*))^*T*^ *null*(*A*) = 0. Additionally, (*A*^+^***b***(*t*))^*T*^ *A*^+^***b***(*t*) does not change with **γ**(*t*), so that its removal from the optimization problem does not change the result. Thus, it is of note that Equation (9) is equivalent to the quadratic program of Equation (8).

Other optimization problems could be formulated, but the interesting challenge is that it is not really known what “optimality” means for the fluxes in a biological system or organism. Optimal solutions, with respect to various criteria, could be suggested, but whether these solutions are compatible with additional information about the functional form or about effectors of fluxes needs to be tested for specific problems. A later section examines the minimum-energy solution for a realistic biological system and indeed challenges the validity of this particular solution to some degree. This discussion shows that optimization, which at this stage does not assume any functional form for the fluxes, may lead to fluxes that can are questionable. At the same time, these optimal solutions can be utilized as starting points for approaching solutions that appear to be biologically meaningful.

### Illustration example: the biosynthetic pathway of aspartate-derived amino acids in the plant *arabidopsis thaliana*

After characterizing a feasible set of fluxes, optimizing the parameters for these fluxes yields a reasonable default solution. Nonetheless, accounting additionally for generally expected features of fluxes can lead to more biologically relevant flux sets. Such generic features may include knowing that a certain flux is a function of only one variable, i.e., its substrate. Another piece of generic information could be that, when a substrate of a flux is zero, the flux has to equal zero as well. These types of constraints are illustrated below with a specific example from the literature, namely the biosynthetic pathway of aspartate-derived amino acids in the plant *Arabidopsis thaliana* (Curien et al., 2009). In reference to the lead author of a model of this system, we will call it the “Curien” model. Since the complete model and the fluxes are known, the pathway system constitutes a good test case. The Gamma-trajectory for the Curien model will be plotted, the criterion of non-negativity and its implication in Gamma-space will be investigated and determined, and the result of optimization will be studied and compared to the original fluxes. Finally, auxiliary methods of flux improvement will be suggested.

#### Identification of flux trends

**Figure 4:**
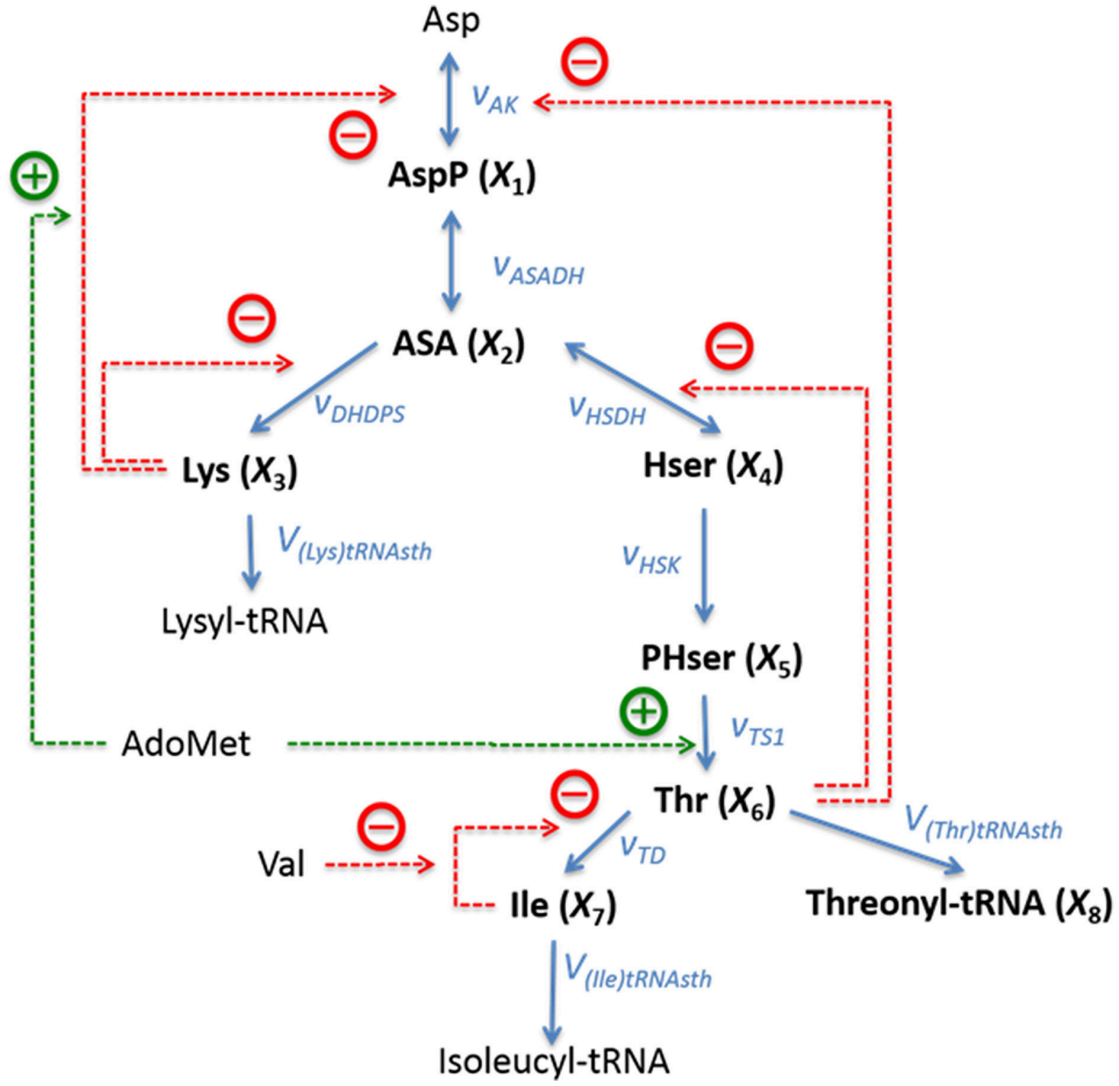
**Metabolic reaction network of the biosynthesis of aspartate-derived amino acids in *Arabidopsis thaliana***. Asp, L-Aspartate; AspP, L-Aspartate-4-phosphate; ASA, L-Aspartate-semialdehyde; Lys, L-Lysine; Hser, Homoserine; PHser, O-Phospho-L-homoserine; AdoMet, S-Adenosylmethionine, Thr, L-Threonine; Ile, L-Isoleucine; Val, L-Valine. Lysyl-tRNA and Isoleucyl-tRNA are shown here as end products, but they are not explicitly included in the model. Adapted from Curien et al. (2009).

The pathway of biosynthesis of aspartate-derived amino acids is responsible for the distribution of the carbon influx into the synthesis of threonine, lysine, methionine, and isoleucine (Figure 4). The original kinetic model (Curien et al., 2009) was constructed based on *in vitro* kinetic measurements, assuming generalized functional forms of the fluxes in the tradition of Michaelis and Menten. The model contains seven dependent variables, namely, *X*_1_ = [aspartyl-phosphate], *X*_2_ = [aspartate semialdehyde], *X*_3_ = [lysine], *X*_4_ = [homoserine], *X*_5_ = [phosphohomoserine], *X*_6_ = [threonine], and *X*_7_ = [isoleucine]. Additionally we consider the output variable *X*_8_ = [threonyl-tRNA].

This specific example is well-suited as an illustration of the proposed techniques of flux identification, because it is representative and of moderate complexity, and because its details are fully known, which facilitates method development and multiple diagnoses of problems that are likely to arise.

The equations for the model

(10) $$\begin{array}{c} \begin{array}{l} \frac{dX_{1}}{dt}=v_{AK}-v_{ASADH}\\ \frac{dX_{2}}{dt}=v_{ASADH}-v_{DHDPS}-v_{HSDH}\\ \frac{dX_{3}}{dt}=v_{DHDPS}-v_{\left(Lys\right)tRNAsth}\\ \frac{dX_{4}}{dt}=v_{HSDH}-v_{HSK}\\ \frac{dX_{5}}{dt}=v_{HSK}-v_{TS1}\\ \frac{dX_{6}}{dt}=v_{TS1}-v_{TD}-v_{\left(Thr\right)tRNAsth}\\ \frac{dX_{7}}{dt}=v_{TD}-v_{\left(Ile\right)tRNAsth}\\ \frac{dX_{8}}{dt}=v_{\left(Thr\right)tRNAsth} \end{array} \end{array}$$

are directly taken from the original article. The functional forms of the fluxes are presented in Equation (11):

(11) $$\begin{array}{c} \begin{array}{l} \;v_{AK1}=\left[AK1\right]\cdot \frac{5.65-1.6[AspP]}{1+{\left([Lys]/\left(\frac{550}{1+\left[AdoMet\right]/3.5}\right)\right)}^{2}}\\ \;v_{AK2}=\left[AK2\right]\cdot \frac{3.15-0.86[AspP]}{1+{\left([Lys]/22\right)}^{1.1}}\\ \;v_{AKI}=\left[AKI-HSDH\;I\right]\cdot \frac{0.36-0.15[AspP]}{1+{\left([Thr]/124\right)}^{2.6}}\\ \;v_{AKII}=\left[AKII-HSDH\;II\right]\cdot \frac{1.35-0.22[AspP]}{1+{\left([Thr]/109\right)}^{2}}\\ \;v_{AK}=v_{AK1}+v_{AK2}+v_{AKI}+v_{AKII}\\ \;v_{ASADH}=\left[ASADH\right]\cdot \left(0.9[AspP]-0.23[ASA]\right)\\ \;v_{HSDH\;I}=\left[AKI-HSDH\;I\right]\cdot 0.84\cdot \\ \;\left(0.14+\frac{0.86}{1+\left[Thr\right]/400}\right)\\ \;v_{HSDH\;II}=\left[AKII-HSDH\;II\right]\cdot 0.64\cdot \\ \;\left(0.25+\frac{0.75}{1+\left[Thr\right]/8500}\right)\\ \;v_{HSDH}=v_{HSDH\;I}+v_{HSDH\;II}\\ \;v_{DHDPS1}=\left[DHDPS1\right]\cdot [ASA]\cdot \frac{1}{1+{\left([Lys]/10\right)}^{2}}\\ \;v_{DHDPS2}=\left[DHDPS2\right][ASA]\cdot \frac{1}{1+{\left([Lys]/33\right)}^{2}}\\ \;v_{DHDPS}=v_{DHDPS1}+v_{DHDPS2}\\ v_{\left(Lys\right)tRNAsth}=V^{AaRS}\cdot \frac{\left[Lys\right]}{25+[Lys]}\\ \;v_{HSK}=\left[HSK\right]\cdot \frac{2.8[Hser]}{14+[Hser]}\\ \;v_{TS1}=\left[TS1\right]\cdot \\ \;\frac{\left(\frac{0.42+3.5{\left[AdoMet\right]}^{2}/73}{1+{\left[AdoMet\right]}^{2}/73}\right)[PHser]}{\left[\frac{250\left(\frac{1+\left[AdoMet\right]/0.5}{1+\left[AdoMet\right]/1.1}\right)}{1+\frac{{\left[AdoMet\right]}^{2}}{140}}\right]\left(1+\frac{\left[P_{i}\right]}{1000}\right)+[PHser]}\\ v_{\left(Thr\right)tRNAsth}=V^{AaRS}\cdot \frac{\left[Thr\right]}{100+[Thr]}\\ \;v_{TD}=\left[TD\right]\cdot \frac{0.0124[Thr]}{1+{\left([Ile]/\left(30+\frac{74[Val]}{610+\left[Val\right]}\right)\right)}^{3}}\\ v_{\left(Ile\right)tRNAsth}=V^{AaRS}\cdot \frac{\left[Ile\right]}{20+[Ile]} \end{array} \end{array}$$

Equation (10) can equivalently be written in vector form as shown in Equation (7), namely as

(12) $$\begin{array}{c} \frac{dX}{dt}=\dot{X}=Av \end{array}$$

where ***v*** and *A* are the corresponding vector of reaction rates (i.e., fluxes) and the stoichiometric matrix, respectively. For the Curien model, they are shown in Equations (13) and (14):

(13) $$\begin{array}{l} v=[v_{AK},v_{ASADH},v_{HSDH},\;v_{DHDPS},\;v_{\left(Lys\right)tRNAsth},v_{HSK},v_{TS1},\\ \;{v_{\left(Thr\right)tRNAsth},v_{TD},v_{\left(Ile\right)tRNAsth}]}^{T}\\ \;={\left\{\;v_{1},v_{2},\;v_{3},v_{4},\;v_{5},\;v_{6},v_{7},\;v_{8},v_{9},\;v_{10}\right\}}^{T} \end{array}$$

(14) $$\begin{array}{c} A=\left(\begin{array}{cccccccccc} \text{1} & \text{-1} & 0 & 0 & 0 & 0 & 0 & 0 & 0 & 0\\ 0 & \text{1} & \text{-1} & \text{-1} & 0 & 0 & 0 & 0 & 0 & 0\\ 0 & 0 & 0 & \text{1} & \text{-1} & 0 & 0 & 0 & 0 & 0\\ 0 & 0 & \text{1} & 0 & 0 & \text{-1} & 0 & 0 & 0 & 0\\ 0 & 0 & 0 & 0 & 0 & \text{1} & \text{-1} & 0 & 0 & 0\\ 0 & 0 & 0 & 0 & 0 & 0 & \text{1} & \text{-1} & \text{-1} & 0\\ 0 & 0 & 0 & 0 & 0 & 0 & 0 & 0 & \text{1} & \text{-1}\\ 0 & 0 & 0 & 0 & 0 & 0 & 0 & \text{1} & 0 & 0 \end{array}\right) \end{array}$$

#### Gamma-trajectory of the curien model

The fluxes and metabolite concentrations for this system are known, which allows us to plot the “true” Gamma-trajectory in the Gamma-space representation vs. time:

(15) $$\begin{array}{c} v\left(t\right)=A^{+}b\left(t\right)+null\left(A\right)\gamma \end{array}$$

Here,

(16) $$\begin{array}{l} null\left(A\right)=\left[vec_{1},vec_{2}\right]\\ \;={\left[\begin{array}{cc} \begin{array}{cc} \text{0}.\text{5374} & \text{0}.\text{5374} \end{array} & \begin{array}{ccc} \begin{array}{ccc} \text{0}.\text{1162} & \text{0}.\text{4212} & \text{0}.\text{4212} \end{array} & \begin{array}{ccc} \text{0}.\text{1162} & \text{0}.\text{1162} & 0 \end{array} & \begin{array}{cc} \text{0}.\text{1162} & \text{0}.\text{1162} \end{array} \end{array}\\ \begin{array}{cc} \text{0}.\text{0534} & \text{0}.\text{0534} \end{array} & \begin{array}{ccc} \begin{array}{ccc} \text{0}.\text{3914} & \text{-0}.\text{3380} & \begin{array}{ccc} \text{-0}.\text{3380} & \text{0}.\text{3914} & \text{0}.\text{3914} \end{array} \end{array} & 0 & \begin{array}{cc} \text{0}.\text{3914} & \text{0}.\text{3914} \end{array} \end{array} \end{array}\right]}^{T} \end{array}$$

spans the null space of *A*. This solution is easily found, as *null*(*A*) is a MATLAB command that returns these two orthonormal vectors. **γ**(*t*) = [γ_1_(*t*), γ_2_(*t*)]^*T*^ is the vector of coefficients associated with *null*(*A*). With this information, the two-dimensional Gamma-space can be explored instead of the feasible subset of the 10-dimensional space of fluxes.

For each time point *t*, the gamma coefficients can be calculated by projecting ***v**_**n**ull_* (*t*) = ***v***(*t*) − *A*^+^***b*** (*t*) onto the vectors *vec*_1_ and *vec*_2_. The result is equivalent to the dot product of *null*(*A*) and ***v***(*t*), since *A*^+^***b***(*t*) is orthogonal to the null space and the dot product is zero.

**Figure 5:**
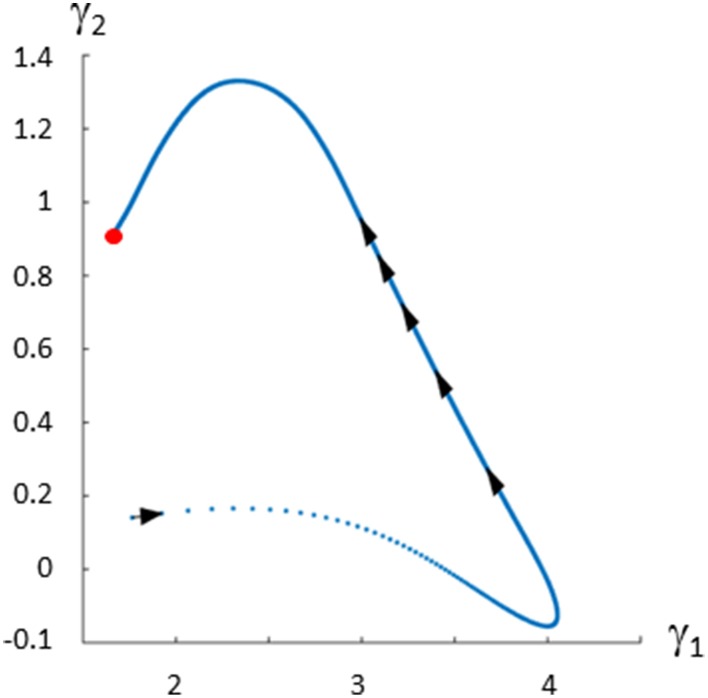
**Gamma-trajectory for the Curien model (Curien et al., 2009)**. The spacing of arrows shows the progression of time. The steady state is shown in red.

Figure 5 shows the trajectory starting at time zero and ending at steady state shown with a red dot.

#### Feasible solutions

**Figure 6:**
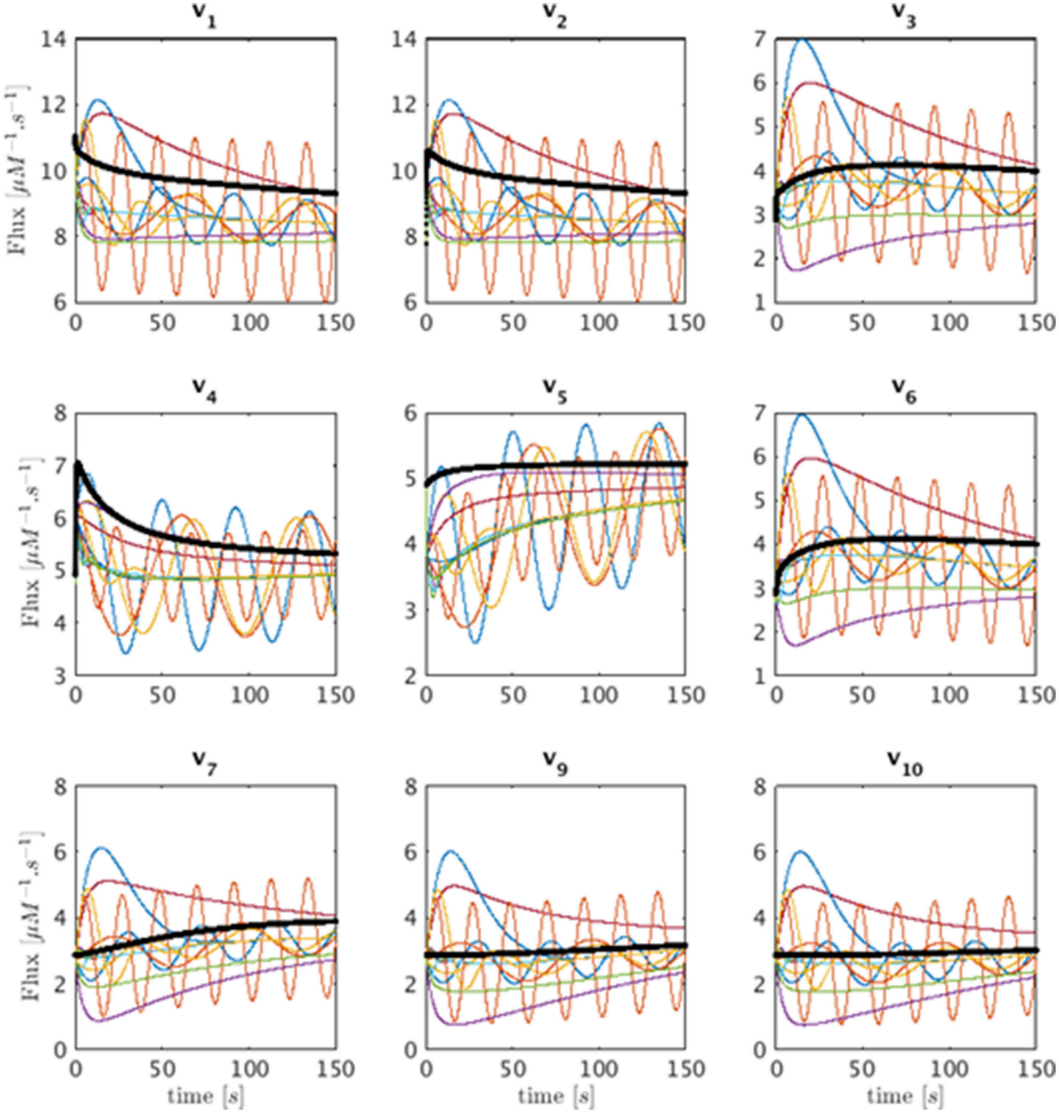
**Sets of feasible solutions for each flux *v*_1_ to *v*_7_ and *v*_9_ to *v*_10_ is shown in each panel**. For visualization purposes, the actual flux from the model is shifted to have the same initial value as the simulated fluxes and is superimposed as a thick black line for comparison.

Similar to the introductory example, this model permits an infinite number of solutions, which may be quite different. Some of these feasible solutions can be generated with a Monte-Carlo simulation by starting at some initial point in the Gamma-space and computing a phase-plane trajectory according to the linear state-space model of $\dot{\gamma }\left(t\right)=B\gamma \left(t\right)$, as before. The resulting trajectories exhibit a variety of different dynamical characteristics for the fluxes. Panels 1–9 of Figure 6 show in multiple colors a selection of feasible solutions for fluxes *v*_1_ through *v*_10_, with the exception of the output flow *v*_8_. Flux *v*_8_ is not shown since it belongs to the only full rank subset of the system and is fully determined by numerically differentiating *X*_8_. The thin lines representing these solutions are superimposed on the actual flux (black), which is known from the model. It is evident that some of the inferred fluxes are similar to the actual fluxes, but that many are not even qualitatively of the same shape. In order to facilitate easier comparisons, the fluxes shown are shifted so that their initial values match. Interestingly, the inferred fluxes show different behaviors ranging from monotonic to various oscillatory shapes. One should note that these feasible solutions are typical examples if we assume a trajectory from a linear state-space solution but that they by no means represent all the possible trends.

**Figure 7:**
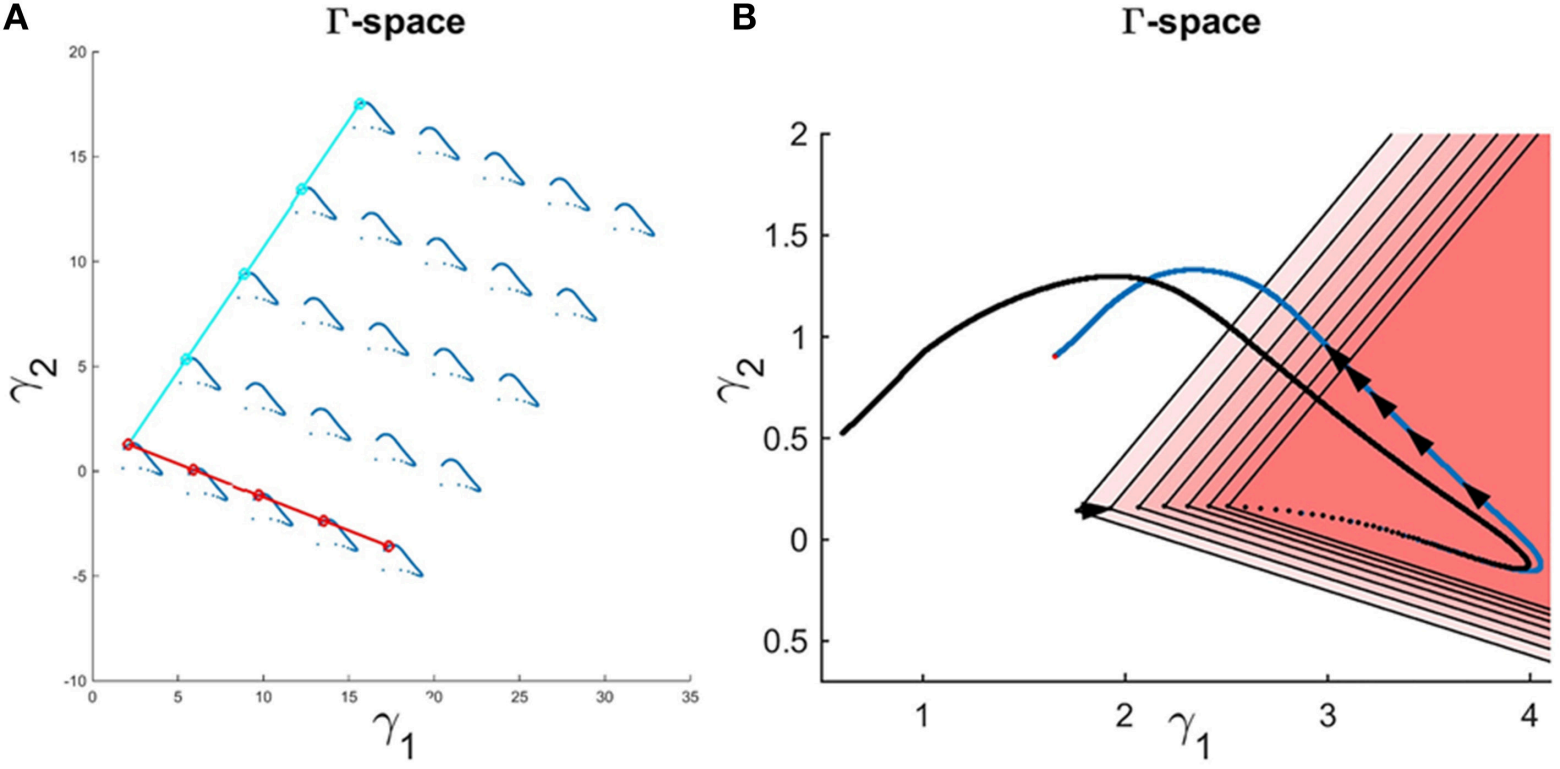
**(A)** Adding a constant amount to the fluxes in Set 1 for all time points shifts the Gamma-trajectory along the dark red line without any change in the concentration profiles for all metabolites. Similarly, adding a constant amount to the fluxes in Set 2 for all time points shift the Gamma-trajectory along the cyan line without any change in the concentration profiles for all metabolites. **(B)** The Gamma-trajectory of the Curien model is depicted in blue color. The black arrowheads shown halfway through the blue curve are equally spaced in time. The open red triangles show the subset of the Gamma-space where the corresponding flux set is non-negative at each point in time. Only the first seven triangles are shown for illustration purposes. The black doted curve shows the corners of these open triangles for different time points. We will later see that, for the Curien model example, this curve is the same as the minimum-energy curve as described before. Interestingly the blue and black curves are overlapping in the beginning but then diverge.

An interesting observation is that one may add an equal value to each flux in *Set* 1 = {*v*_1_, *v*_2_, *v*_4_, *v*_5_} and/or *Set* 2 = {*v*_1_, *v*_2_, *v*_3_, *v*_6_, *v*_7_, *v*_9_, *v*_10_} without a change in the metabolite concentration profiles. The reason is that these shifts cancel out in the original differential equations (Equation 10) and $\dot{X}\left(t\right)$ (*t*) therefore stays the same. Figure 7A demonstrates that the shape of the Gamma-trajectory (Figure 5) can be shifted along the red line if one adds different positive constant amounts to *Set* 1 and along the cyan line if one adds different positive constant amounts to *Set* 2. Of course, shifts in both directions are admissible as well. One could also pick negative constant values as long as the fluxes stay positive. This way, the entire Gamma-space can be spanned. This is an equivalent, and perhaps more comprehensible, explanation of the two degrees of freedom for this pathway. As an alternative to constant shifts, it is even admissible to add the same function of time to all fluxes in the sets.

#### Admissible subset of the gamma-space: the subspace of non-negative fluxes

For each time point *t*, we determine the set of **γ**'s for which the corresponding ***v***(*t*) consists entirely of non-negative fluxes. Recalling Equation (6), the feasible space here is an intersection of 10 half spaces characterized by the following set of inequalities:

(17) $$\begin{array}{c} A^{+}\left(i,:\right)b\left(t\right)+{\text{γ}}_{1}vec_{1,i}+{\text{γ}}_{2}vec_{2,i}\geq 0\;i=1,\;2,\cdots ,\;10 \end{array}$$

Here, *A*^+^(*i*, :) denotes the *i*^*th*^ row of the 10 × 8 Moore-Penrose pseudo-inverse matrix.

In this example, only two out of the total of 10 inequalities happen to be active inequalities, which results in a feasible subspace in the shape of an open triangle. One should note, however, that ***b****(t)* changes with time, so that there is a new open triangle for each time point. Expressed differently, the feasible region resulting in non-negative flux sets varies with each time point. Figure 7B exhibits the first seven of these open triangles in different shades of red. There is one such triangle for each time point; the triangles are not shown for the following time points to avoid over-population of the plot.

The corners of these open triangles are shown as black dots, which lie on a curve. The blue curve shows the actual Gamma-trajectory of Figure 5. One interesting observation is that, for the initial time points, the two curves (“true” and inferred) coincide. For later time points, the blue curves lie inside the corresponding open triangle of non-negative solutions.

Any continuous trajectory whose points fall inside these non-negative open triangles for all time points is a feasible flux profile.

#### Minimum-energy flux set

Searching the feasible solutions for the set of flux profiles that minimize the sum of squared flux norms for all time points results in the minimum-energy flux. This procedure is equivalent to solving the quadratic programming of Equation (9) and results in the same flux profile as solving the linear programming of Equation (7). For the case of the Curien model, both of these methods yield the same set of fluxes as the corner solution introduced in the previous section. This solution is also equivalent to the result of a non-negative least-squares optimization problem performed in MATLAB.

**Figure 8:**
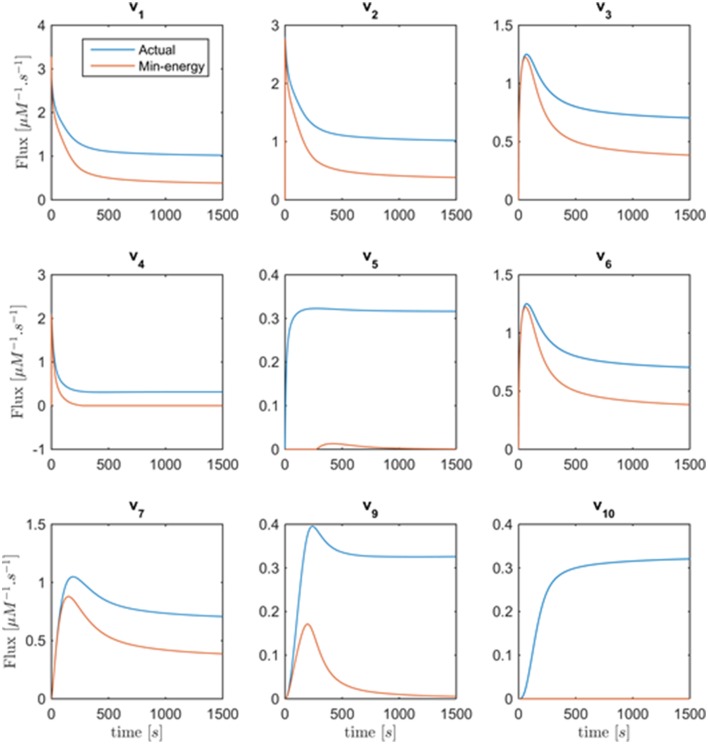
**Fluxes *v*_1_ to *v*_10_ with the exception of *v*_8_ are plotted vs. time**. Curves in red are the min-energy fluxes, while the blue curves show the actual fluxes of the Curien model. Flux *v*_8_ is not shown because it belongs to the full-rank subset of the system and can be recovered exactly from the data.

Figure 8 shows the minimum energy flux profiles plotted vs. time (depicted in red) together with the actual fluxes of the Curien model (blue). The two solutions are quite different, although they both match the metabolite data perfectly. The next sections introduce strategies to alleviate this discrepancy. One should note that the computed solution is actually “cheaper” than the Curien model, as all fluxes have lower magnitudes; whether it is “better” or “worse” than the Curien model cannot be said, because we do not know the correct criteria.

#### Generally expected features regarding fluxes can restrict the feasible space further

**Figure 9:**
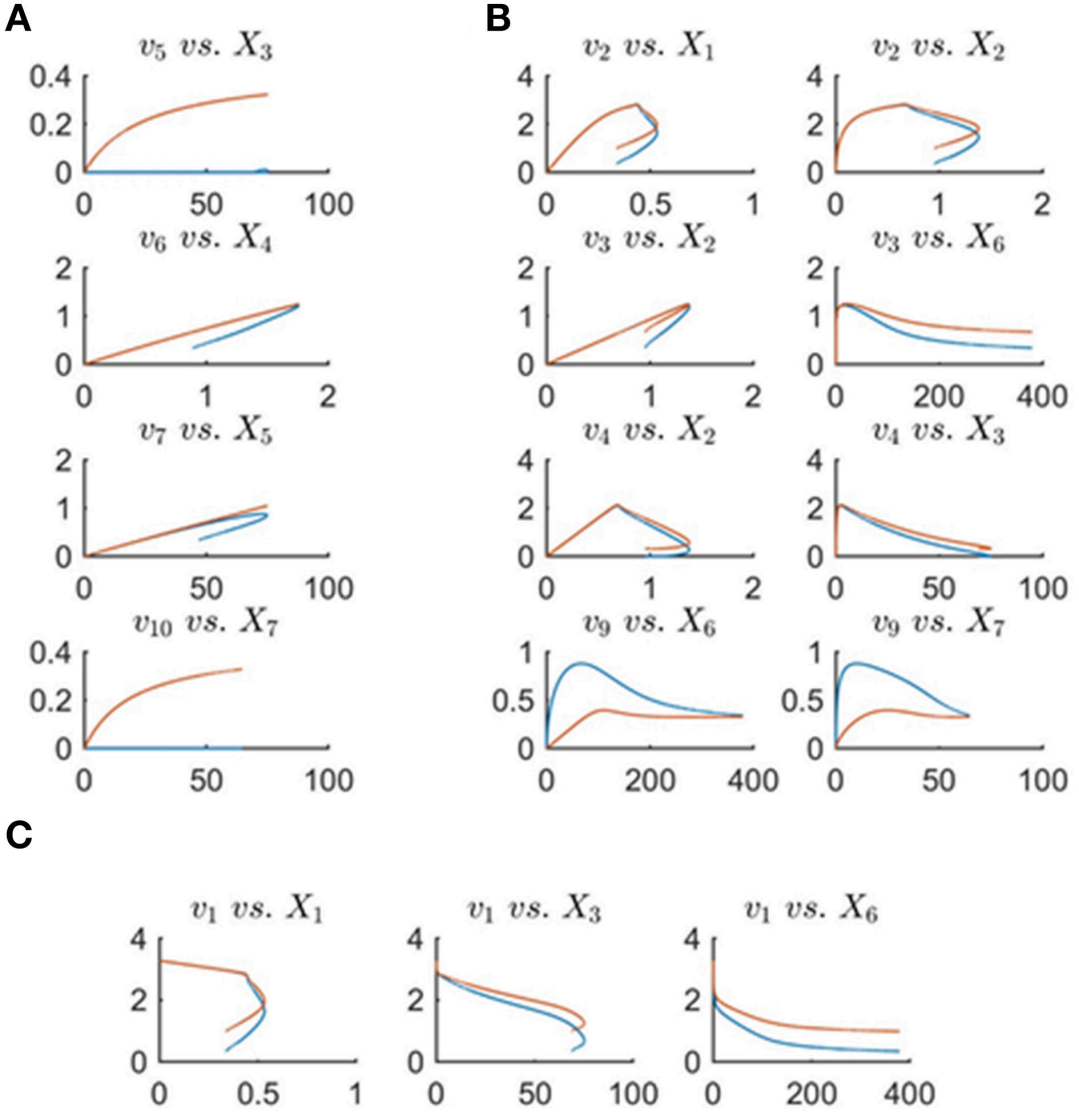
**Panel (A) shows one-substrate fluxes of the system plotted against their substrate concentrations**. Note that fluxes *v*_6_ and *v*_7_ exhibit a folding-over phenomenon. Panel **(B)** depicts the plots of fluxes that have two effectors (substrates or regulators) vs. each variable separately. Panel **(C)** shows flux *v*_1_ vs. its participating variables. In all plots, the actual fluxes, as known from the original model, are plotted in red, while blue shows the min-energy fluxes.

General expectations regarding metabolic fluxes may constrain the feasible flux profiles. To assess these expectations, it is useful to plot the fluxes against their substrates and modulators rather than against time, as was done before. Figure 9 shows all actual fluxes plotted against their substrates and effectors in blue, super-imposed on the min-energy fluxes vs. their substrates and effectors in red. Fluxes *v*_5_, *v*_6_, *v*_7_, and *v*_10_ are known to be functions solely of their corresponding substrates, while fluxes *v*_2_, *v*_3_, *v*_4_, and *v*_9_ have two substrates/regulators, and *v*_1_ has three. Closer inspection of these plots reveals that the plots of *v*_6_ vs. *X*_4_ and *v*_7_ vs. *X*_5_ show a behavior that is not consistent with a true mathematical function, namely a folding-over (Figure 9A). For example, if the concentration of *X*_4_ is 1.2μM, flux *v*_6_ may take two values, and therefore cannot be a function in the mathematical sense. Assuming that we know that no other variables affect this flux, this folding-over phenomenon is not acceptable.

To ameliorate this problem, one may remove or cut the folded-over section. Specifically, for the time points corresponding to folded-over values, we let *v*_6_ take values according to the top branch. This is allowable, as the upper branch is a feasible solution. Using this technique, *v*_6_(*t*) becomes uniquely determined and can be considered an identified flux. Subsequently, a new min-energy response can be computed with exactly the same methods as before, but with only one degree of freedom left.

**Figure 10:**
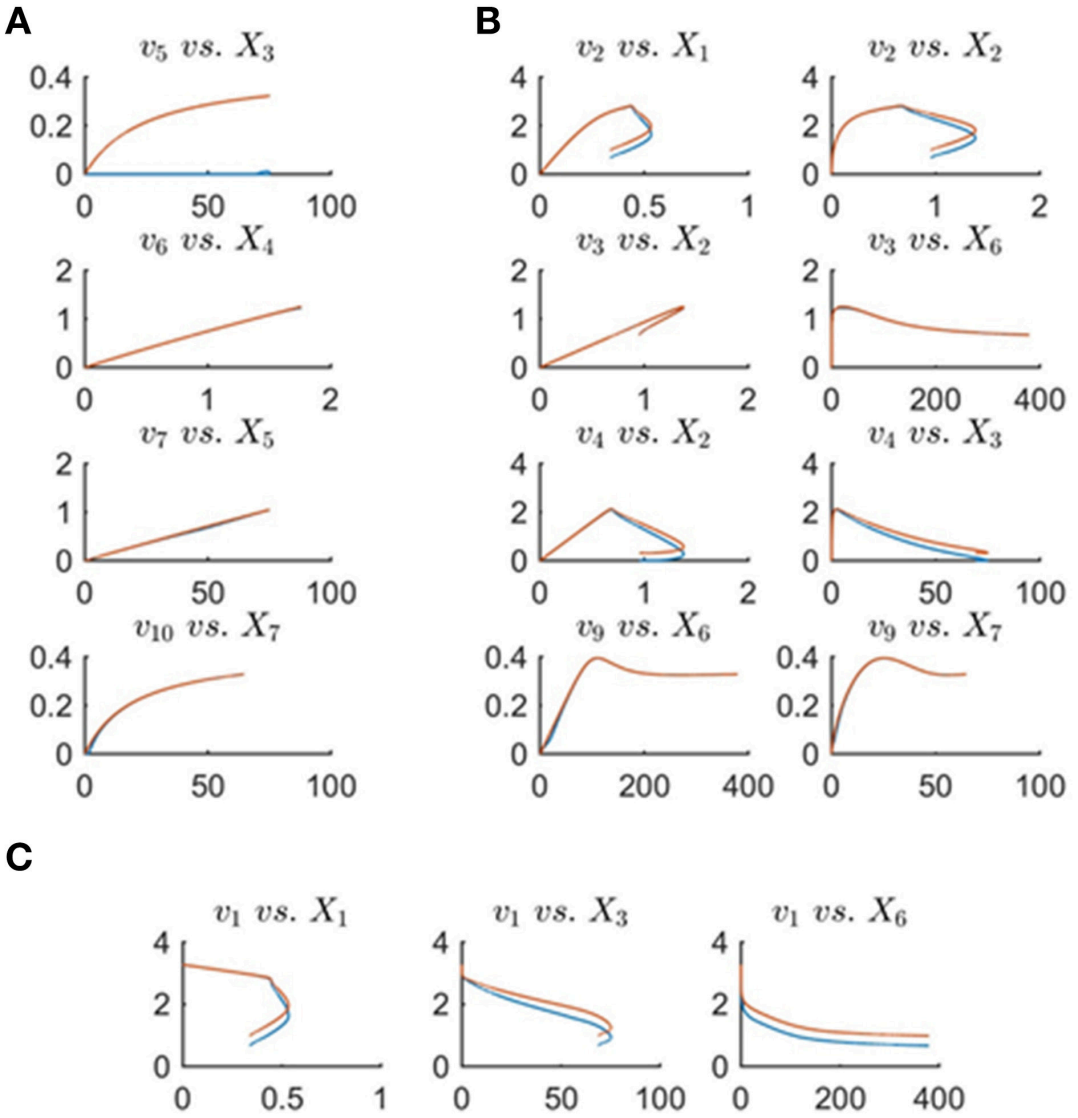
**This figure shows the same plots as in Figure 9 with the difference that the plots in blue are the min-energy fluxes after fixing the folding-over problem**. Panel **(A)** shows the one-variable fluxes vs. their substrates. Panel **(B)** depicts the plots of fluxes that have two effectors (substrates or regulators) vs. each variable separately. Panel **(C)** shows flux *v*_1_ vs. its participating variables. In all plots, the actual fluxes, as known from the original model, are plotted in red, while blue shows the min-energy fluxes after resolving the folding-over problem.

Figure 10 depicts the same plots as in Figure 9 after removing the folding-over phenomenon. Interestingly, all fluxes in *Set* 2 = {*v*_1_, *v*_2_, *v*_3_, *v*_6_, *v*_7_, *v*_9_, *v*_10_}, as introduced before, are now fixed and almost equivalent to the actual fluxes. This means that the number of degrees of freedom has decreased to 1 after incorporating the information that one of the fluxes is a function of one variable only. The discrepancy between fluxes in *Set* 1 = {*v*_1_, *v*_2_, *v*_4_, *v*_5_} remains unsolved, and there is no other folding-over among the one-variable fluxes.

A caveat of the strategic step above is our assumption that some of the fluxes only depend on their substrates. Such an assumption is of course not always valid, but the more we learn about metabolism the more we will be able to rely on solid information. To validate such an assumption, one might use a step-wise scheme of testing additional variables as modulators (Marino and Voit, 2006). By the same token, the proposed methods may actually point to regulatory signals that had been unknown or overlooked (Dolatshahi et al., 2016a,b). One notes that this issue is a challenge for any estimation or identification strategy.

**Figure 11:**
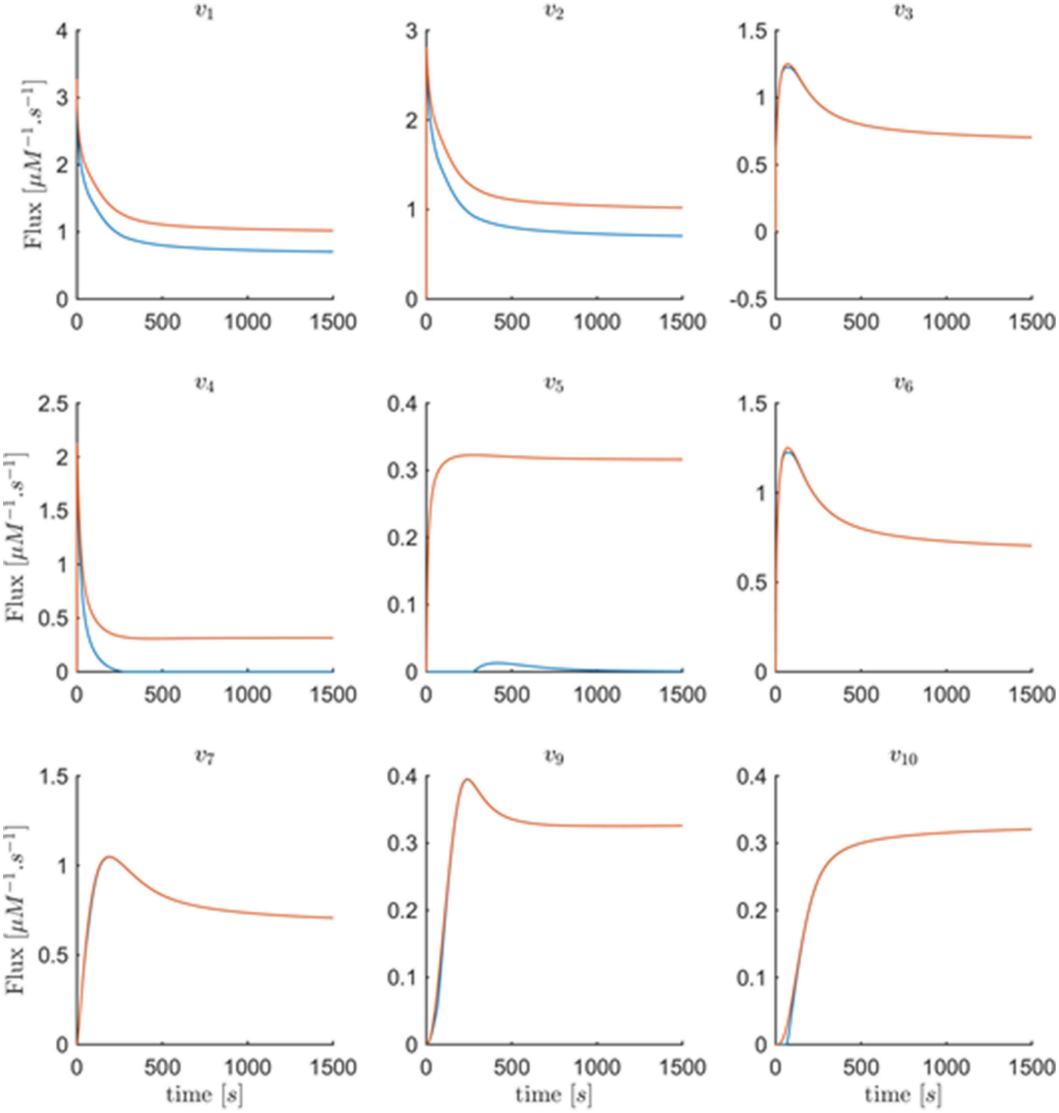
**Fluxes *v*_1_ to *v*_10_ with the exception of *v*_8_ are plotted vs. time**. The red curves are the min-energy fluxes after solving the folding-over problem, while the blue curves show the actual fluxes. It is evident that the fluxes *v*_3_, *v*_6_, *v*_7_, v_9_, *v*_10_ are almost identical and overlapping and that our method has recovered these fluxes.

In order to recover the fluxes in *Set* 1, additional information is needed. First, one could assume that all fluxes in this set are shifted by the same value. If this value were chosen as about 0.3, one can imagine from Figure 11 that the fluxes become very similar to the fluxes in the original model. Second, suppose it was known that, for instance, *v*_5_ is well-modeled as a Michaelis–Menten rate function and the corresponding kinetic parameters *K*_*M*_ and *V*_*max*_ could be extracted from the literature. Then one could find *v*_1_, *v*_2_, *v*_4_ by the following simple procedure: Determine the shift function $f_{shift}\left(t\right)\;=\frac{V_{max}\;X_{3}\left(t\right)}{k_{m}+X_{3}\left(t\right)}-v_{5-min}\left(t\right)$ and add it to the rest of fluxes in *Set* 1 to find the actual fluxes; thus, *v*_*j*_(*t*) = *v*_*j*−*min*_(*t*) + *f*_*shift*_(*t*), *j* ∈ {1, 2, 4}. Indeed, if the Michaelis–Menten function is implemented with Curien's parameter values, the entire system is perfectly recouped (result not shown). Having said that, there is no objective argument against the fluxes in Figure 11, except possibly that *v*_5_ is essentially 0 for the first 250 time units, and then becomes slightly non-monotonic, which might not be realistic. At the same time, the computed fluxes are of lower magnitude than those in the Curien model. As a third alternative, one could independently determine one of the fluxes in *Set* 1, for example as a power-law function, as it was demonstrated elsewhere (Iwata et al., 2013), and then compute all other fluxes of the set.

## Discussion

### Extension of dfe toward pathways with incomplete information

In many practical scenarios, some of the data are missing, and/or some of the fluxes cannot be determined fully even with the techniques described in the previous sections. If so, the need arises for additional strategies that make maximal use of DFE's capabilities and diagnostic features (Voit, 2009; Chou and Voit, 2012; Iwata et al., 2013), along with random search and global optimization techniques.

**Figure 12:**
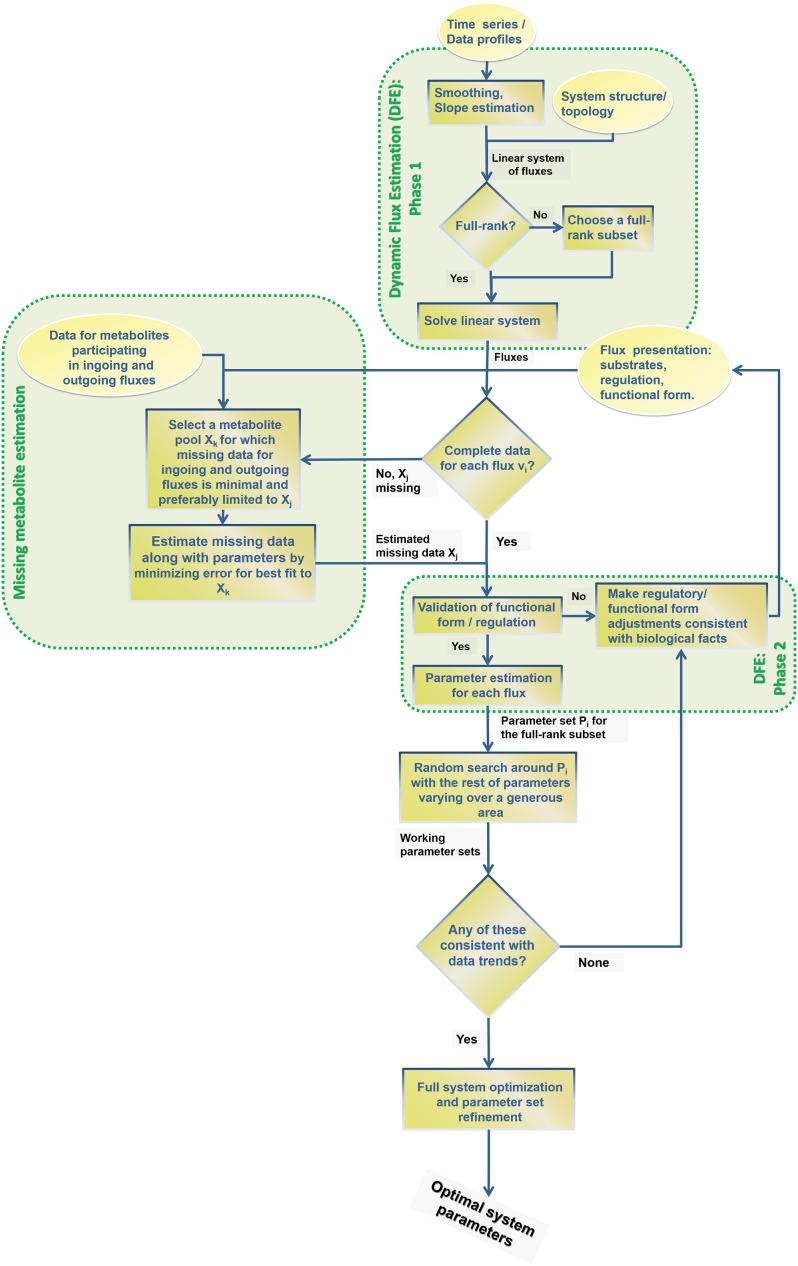
**Step-by-step procedure for the proposed extension of dynamic flux estimation (DFE)**.

Because data are seldom ideal, this section discusses a rather generic, multi-step strategy that takes advantage of the diagnostic and computational benefits that DFE offers, and augments them with auxiliary methods and global optimization approaches toward a full-system parameterizations (Figure 12). These procedures were recently used for the construction of a complex model of the highly regulated glycolytic pathway of *Lactococcus lactis* from NMR data (Dolatshahi et al., 2016a,b) where, due to missing data and other features of the data, the estimation of parameters was not straightforward.

The first step of this strategy consists of identifying full rank subsets of fluxes within the system (see flux estimation module in Figure 12), if that is possible. For instance, the *Arabidopsis* example allowed us to identify *Set* 2 as well as the flux *v*_8_.

Suppose now that data for one or more of the variables are missing. If so, the “missing metabolite estimation module” in Figure 12 is used (see also Voit, 2009). The goal is to infer flux information from near-by metabolites or at least to constrain the parameters of this flux for the following steps of a randomized search and global full system parameterization. This module involves an optimization task, which ideally yields valuable information regarding the likely profile of the missing data. The first step in this module consists of selecting a metabolite pool that is close to the missing data, includes a concentration profile, and has influxes and effluxes that are at least partially characterized. As an example, assume time series data for lysine (*X*_3_) were missing in the Curien model. The idea is to infer the missing data from other metabolites and/or identifiable fluxes. For instance, information regarding lysyl-tRNA could provide valuable hints regarding *V*_(*Lys*)*tRNAsth*_: namely, one could assume a power-law or Michaelis–Menten function to infer *X*_3_ from the data for the accumulation of lysyl-tRNA. In this particular case, the computation of *X*_3_ from *V*_(*Lys*)*tRNAsth*_ at different time points would actually be quite simple, as both functional formats can be transformed into linear equations.

If such an inference is not feasible, other biological information is needed and must be supplied on a case-by-case basis. For instance, biological arguments may provide clues regarding amounts that might reasonably be added to formerly identified flux sets. In some cases, measurements fall below the detection limit, so that no numerical data are available, although the biology of the system mandates that the concentrations are not zero. The detection limit, mass conservation, and possibly other considerations can serve as useful constraints for the optimization algorithm. The output of this module thus consists of substitutes for some of the missing data profiles, along with their associated parameter values. In other parts of the workflow, these are treated like experimental data.

The “validation of functional form and regulation” step assesses the appropriateness of the functional formats for the flux representations. A first and obvious criterion is the quality of the fit, which is necessary, although not sufficient (Voit, 2011). A second criterion is the detection or lack of “runs in residuals” (Draper and Smith, 1981). If no appropriate format and parameterization can be found, it is quite probable that important components of the pathway are missing from the model. An example is the situation where a flux decreases with increasing, reasonable substrate concentrations. Such a trend is counterintuitive and may suggest that a regulator is missing from the model. If so, DFE can possibly help identify what shape the dynamic trend of the regulator must have to remedy the discrepancy. A scan of the dynamics of all variables in the model may even identify candidates, although such inferences are still to be tested experimentally. Examples of this situation are presented elsewhere (Dolatshahi et al., 2016a,b).

Beyond the quality of fit and run test, no true validation is possible, because the fluxes are unknown. Even so, the “validation of functional form and regulation” step ensures reasonableness and flags fluxes that are computed as negative, exhibit unduly high magnitudes, or are apparently lacking important contributing variables.

### Assessment of the inferred fluxes and their parameters

Once the functional forms and regulations are considered satisfactory and the corresponding parameters are estimated, it is necessary to test whether the estimated parameter set is essentially unique or whether substantially different solutions exist. This identifiability and sloppinenss step (e.g., Gutenkunst, 2007a,b; Vilela, 2009; Raue, 2013; Villaverde and Banga, 2013; Tafintseva, 2014; Tönsing et al., 2014) is particularly pertinent if the data are noisy or some of the data were not measured but inferred in earlier steps. This global analysis often utilizes Monte Carlo simulations, in which a large-scale random search is anchored in the estimated, optimal parameter set {*P*_*i*_}, which serves as the starting point for the global optimization. The differences in the sets of newly estimated parameter values for each flux and each experiment are collectively used to determine admissible ranges for the parameters of the system and starting values for global optimization. This last estimation step entails a combination of different optimization techniques, which may begin with evolutionary (genetic) algorithms that provide coarse solutions and are followed up with steepest descent algorithms that refine these solutions. The objective function for this purpose is the usual sum of squared errors over all time points, metabolites, and datasets, but may also include a penalty for metabolite concentrations that were inferred rather than directly measured. The ideal outcome of this step is either an essentially unique model parameterization or a compact ensemble of models with parameter values that permit some flexibility without compromising the data fit.

## Conclusions and outlook

The goal of this article was to extend the utility of DFE to the relatively common scenario where the algebraic system of fluxes is underdetermined or some time series data are missing or incomplete.

Initially, the concept of lower-dimensional representation in the form of a so-called Gamma-space and a Gamma-trajectory was introduced. This representation is especially useful when the number of degrees of freedom is low. Reasonable biological constraints like smoothness over time and non-negativity of fluxes were taken into account to constrain the feasible space even further. In particular, a minimum-energy criterion was considered, and solutions were discarded in which fluxes were not representable by mathematical functions, due to non-uniqueness. The concepts were illustrated with a model of aspartate metabolism in the plant *Arabidopsis*. The minimum-energy flux set did not match the actual flux profiles for this pathway, even though the metabolite data were recouped with a set of fluxes that had lower magnitudes than in the original model. The addition of biologically reasonable constraints reduced the discrepancies. In particular, it was known that a certain flux, *v*_6_, is a function of only its substrate. This knowledge helped us reshape the minimum-energy flux, with the consequence that more than half of the resulting fluxes of the system became identifiable and indeed matched the original flux profile. Additional knowledge—or assumptions—about the fluxes can potentially constrain the feasible space of solutions further and may recover the original flux set. For example, knowing (or assuming) that a certain flux follows a specific functional form can potentially lead to a determination of this flux and decrease the degrees of freedom by one (cf. Iwata et al., 2013).

More generically, it is not always clear what optimality criteria or constraints should be evoked to reduce the feasible set of solutions, where all fit the concentration data exactly. Nonetheless, the identification and characterization of feasible flux sets may lead to a better understanding of the system and possibly aid the design of additional experiments that could effectively fill the gap and recover the true fluxes. Ideally, such experiments should yield data where all (most, or many) variables cover as much of their relevant substrate ranges as possible.

On a complementary trajectory, incomplete or missing data render the direct employment of DFE for the task of parameter estimation impossible. Nonetheless, a mixed strategy of DFE and optimization may alleviate the problem and lead at least to subsets of identified fluxes.

## Author contributions

SD and EV conceived the study. SD performed all analyses. SD and EV interpreted the results and wrote the paper.

### Conflict of interest statement

The authors declare that the research was conducted in the absence of any commercial or financial relationships that could be construed as a potential conflict of interest.
