# Supplementary material for: Identification of Metabolic Pathway Systems
Source: Front Genet. 2016 Feb 10;7:6. doi: 10.3389/fgene.2016.00006 (PMC4748741; doi:10.3389/fgene.2016.00006)
Supplement: Supplementary file 1 [file Supplementarycodes.PDF]

Supplement: Identification of Metabolic Pathway Systems

Sepideh Dolatshahi and Eberhard O. Voit

Computer Codes

Illustration example: Curien model

1. Gamma-plane characterization

The differential equations explaining the Curien model were taken from the Supplements of [Curien_reference] and are shown in *Diff_Curien_sz.m* MATLAB script below:

Diff_Curien_sz.m

```
function dydt = Diff_Curien_sz(t,y,s,z)
dydt=zeros(8,1);
% ---------------------------------------------------------------------- %
%
% This model and the parameters are taken from the Curien paper and rewritten
% with MATLAB syntax. In this model adoption, Vcgs was set to zero since it
% was more than 5 times smaller than the smallest flux and in order to keep
% the degrees of freedom at 2 for better visualization.
% Also please note that two parameters, s and z, are introduced here to show
% that addition of an equal amount (=s) to certain fluxes belonging to Set 1
% and/or an equal amount (=z) to Set 2 does not change the resulting
% metabolite concentrations.
%
% ---------------------------------------------------------------------- %

% ------------------------- parameters ---------------------------------- %
AK1 = 0.25;
AK2 = 0.25;
AKHSDHII = 0.63;
AKHSDHI = 0.63;
ASADH = 11.6;
DHDPS1 = 1.6;
DHDPS2=1.6;
HSK = 4;
TS1 = 7.4;
TD=0.36;
CGS = 0.7;
LKR=0;
AdoMet=20;
Cys=15;
Phosphate=10000;
Val=100;
AK1_kforward_app_exp=5.65;
AK1_kreverse_app_exp=1.57;
AK1_Lys_Ki_app_exp=550;
AK1_AdoMet_Ka_app_exp=3.5;
```

```
AK1_h_exp=2;
AK2_kforward_app_exp=3.15;
AK2_kreverse_app_exp=0.88;
AK2_Lys_Ki_app_exp=22;
AK2_h_exp=1.1;
AKI_kforward_app_exp=0.36;
AKI_kreverse_app_exp=0.10;
AKI_Thr_Ki_app_exp=124;
AKI_h_exp=2;
AKII_kforward_app_exp=1.35;
AKII_kreverse_app_exp=0.38;
AKII_Thr_Ki_app_exp=109;
AKII_h_exp=2;
ASADH_kforward_app_exp=0.9;
ASADH_kreverse_app_exp=0.23;
DHDPS1_k_app_exp=1;
DHDPS1_Lys_Ki_app_exp=10;
DHDPS1_h_exp=2;
DHDPS2_k_app_exp=1;
DHDPS2_Lys_Ki_app_exp=33;
DHDPS2_h_exp=2;
HSDHI_kforward_app_exp=0.84;
HSDHI_Thr_relative_residual_activity_app_exp=0.15;
HSDHI_Thr_relative_inhibition_app_exp=0.85;
HSDHI_Thr_Ki_app_exp=400;
HSDHII_kforward_app_exp=0.64;
HSDHII_Thr_relative_residual_activity_app_exp=0.25;
HSDHII_Thr_relative_inhibition_app_exp=0.75;
HSDHII_Thr_Ki_app_exp=8500;
HSK_kcat_app_exp=2.8;
HSK_Hser_app_exp=14;
TS1_kcatmin_exp=0.42;
TS1_AdoMet_kcatmax_exp=3.5;
TS1_AdoMEt_Km_no_AdoMet_exp=250;
TS1_AdoMet_Ka1_exp=73;
TS1_AdoMet_Ka2_exp=0.5;
TS1_AdoMet_Ka3_exp=1.09;
TS1_AdoMet_Ka4_exp=142;
TS1_Phosphate_Ki_exp=1000;
TS1_h_exp=2;
CGS_kcat_exp=30;
CGS_Cys_Km_exp=460;
CGS_Phser_Km_exp=2500;
CGS_Phosphate_Ki_exp=2000;
TD_k_app_exp=0.0124;
TD_Ile_Ki_no_Val_app_exp=30;
TD_Val_Ka1_app_exp=73;
TD_Val_Ka2_app_exp=615;
TD_h_app_exp=3;
Lys_tRNAS_Vmax=0.43;
Lys_tRNAS_Lys_Km=25;
Thr_tRNAS_Vmax=0.43;
Thr_tRNAS_Thr_Km=100;
Ile_tRNAS_Vmax=0.43;
Ile_tRNAS_Ile_Km=20;
LKR_kcat_exp=3.1;
LKR_Lys_Km_exp=13000;

% --------------------  Flux formulations --------------------------------%
Vak1 =AK1*(AK1_kforward_app_exp - AK1_kreverse_app_exp*y(1))/ ...
    (1+(y(3)/(AK1_Lys_Ki_app_exp/(1+AdoMet/
AK1_AdoMet_Ka_app_exp)))^AK1_h_exp)+s+z;
Vak2 = AK2*(AK2_kforward_app_exp - AK2_kreverse_app_exp*y(1))/ ...
```

```
    (1+(y(3)/ AK2_Lys_Ki_app_exp)^AK2_h_exp);
VakI =AKHSDHI*(AKI_kforward_app_exp - AKI_kreverse_app_exp*y(1))*1/ ...
    (1+(y(6)/ AKI_Thr_Ki_app_exp)^AKI_h_exp);
VakII =AKHSDHII*(AKII_kforward_app_exp - AKII_kreverse_app_exp*y(1))/...
    (1+(y(6)/ AKII_Thr_Ki_app_exp)^AKII_h_exp);
Vasadh = ASADH*(ASADH_kforward_app_exp*y(1)- ASADH_kreverse_app_exp*y(2))+ s+z;
Vdhdps1 = DHDPS1* DHDPS1_k_app_exp *y(2)*(1/(1+(y(3)/DHDPS1_Lys_Ki_app_exp)^
DHDPS1_h_exp))+ s;
Vdhdps2 = DHDPS2* DHDPS2_k_app_exp *y(2)*(1/(1+(y(3)/ DHDPS2_Lys_Ki_app_exp)^
DHDPS2_h_exp));
VhsdhI = AKHSDHI* HSDHI_kforward_app_exp *y(2)*(
HSDHI_Thr_relative_residual_activity_app_exp + ...
    HSDHI_Thr_relative_inhibition_app_exp /(1+y(6)/ HSDHI_Thr_Ki_app_exp))+z;
VhsdhII = AKHSDHII* HSDHII_kforward_app_exp *y(2)*(
HSDHII_Thr_relative_residual_activity_app_exp + ...
    HSDHII_Thr_relative_inhibition_app_exp /(1+y(6)/ HSDHII_Thr_Ki_app_exp));
Vhsk = HSK* HSK_kcat_app_exp *y(4)/( HSK_Hser_app_exp +y(4))+z;
Vts1=TS1*(TS1_kcatmin_exp + TS1_AdoMet_kcatmax_exp *AdoMet^TS1_h_exp
/TS1_AdoMet_Ka1_exp)/ ...
    (1+AdoMet^TS1_h_exp /
TS1_AdoMet_Ka1_exp)*y(5)/((1+Phosphate/TS1_Phosphate_Ki_exp)* ...
    ( TS1_AdoMEt_Km_no_AdoMet_exp *(1+AdoMet/ TS1_AdoMet_Ka2_exp)/...
    (1+AdoMet/ TS1_AdoMet_Ka3_exp))/(1+AdoMet^TS1_h_exp /
TS1_AdoMet_Ka4_exp)+y(5))+z;
% Vcgs= CGS*( CGS_kcat_exp /(1+ CGS_Cys_Km_exp /Cys))*y(5)/...
%     (( CGS_Phser_Km_exp /(1+ CGS_Cys_Km_exp /Cys))*(1+Phosphate/
CGS_Phosphate_Ki_exp)+ y(5));
Vtd = TD*TD_k_app_exp*y(6)/(1+(y(7)/( TD_Ile_Ki_no_Val_app_exp +
TD_Val_Ka1_app_exp *Val/...
    ( TD_Val_Ka2_app_exp +Val)))^ TD_h_app_exp)+z;
VLys_tRNAS = Lys_tRNAS_Vmax *y(3)/(Lys_tRNAS_Lys_Km+y(3))+s;
VThr_tRNAS = Thr_tRNAS_Vmax *y(6)/(Thr_tRNAS_Thr_Km+y(6));
VIle_tRNAS =Ile_tRNAS_Vmax *y(7)/(Ile_tRNAS_Ile_Km+y(7))+z;
Vlkr= LKR*LKR_kcat_exp*y(3)/(LKR_Lys_Km_exp+y(3));

% ------------------  Differential equations -----------------------------%
% dydt(1) = vAK - vASADH ;
dydt(1) = Vak1 + Vak2 + VakI + VakII - Vasadh;
% dydt(2) = vASADH - vDHDPS - vHSDH ;
dydt(2) = Vasadh - Vdhdps1 -Vdhdps2- VhsdhI -VhsdhII ;
% dydt(3) = vDHDPS - vLKR ;
dydt(3) = Vdhdps1 + Vdhdps2 - Vlkr - VLys_tRNAS;
% dydt(4) = vHSDH - vHSK ;
dydt(4) = VhsdhI + VhsdhII -Vhsk;
% dydt(5) = Vhsk  - Vts1 - Vcgs;
dydt(5) = Vhsk  - Vts1;
% dydt(6) = vTS1 - vTD - vThr_tRNAsth;
dydt(6) = Vts1 - Vtd - VThr_tRNAS;
% dydt(7) = Vtd-VIle_tRNAS;
dydt(7) = Vtd-VIle_tRNAS;
% dydt(8) = vThr_tRNAsth;
dydt(8) = VThr_tRNAS;
end
```

The following MATLAB script *Fluxes_Curien_sz.m* shows similar equations explaining the Curien model. The difference is that the output of this file consists of the fluxes and derivatives with respect to time of metabolite concentrations, which will be used later for analysis as well as comparisons.

Fluxes_Curien_sz.m

```
function [fluxes,xdot] = Fluxes_Curien_sz(t,y,s,z)

% ----------------------------------------------------------------------- %
%
% This function outputs the fluxes and xdots for comparison with the result
% of our analyses.
% This model and the parameters are taken from the Curien paper and rewritten
% with MATLAB syntax. In this model adoption, Vcgs was set to zero since it
% was more than 5 times smaller than the smallest flux and in order to keep
% the degrees of freedom at 2 for better visualization.
% Also please note that two parameters, s and z, are introduced here to show
% that addition of an equal amount (=s) to certain fluxes belonging to Set 1
% and/or an equal amount (=z) to Set 2 does not change the resulting
% metabolite concentrations.
%
% ----------------------------------------------------------------------- %

% ------------------------- parameters ---------------------------------- %
AK1 = 0.25;
AK2 = 0.25;
AKHSDHII = 0.63;
AKHSDHI = 0.63;
ASADH = 11.6;
DHDPS1 = 1.6;
DHDPS2=1.6;
HSK = 4;
TS1 = 7.4;
TD=0.36;
CGS = 0.7;
LKR=0;
AdoMet=20;
Cys=15;
Phosphate=10000;
Val=100;
AK1_kforward_app_exp=5.65;
AK1_kreverse_app_exp=1.57;
AK1_Lys_Ki_app_exp=550;
AK1_AdoMet_Ka_app_exp=3.5;
AK1_h_exp=2;
AK2_kforward_app_exp=3.15;
AK2_kreverse_app_exp=0.88;
AK2_Lys_Ki_app_exp=22;
AK2_h_exp=1.1;
AKI_kforward_app_exp=0.36;
AKI_kreverse_app_exp=0.10;
AKI_Thr_Ki_app_exp=124;
AKI_h_exp=2;
AKII_kforward_app_exp=1.35;
AKII_kreverse_app_exp=0.38;
AKII_Thr_Ki_app_exp=109;
AKII_h_exp=2;
ASADH_kforward_app_exp=0.9;
ASADH_kreverse_app_exp=0.23;
DHDPS1_k_app_exp=1;
DHDPS1_Lys_Ki_app_exp=10;
DHDPS1_h_exp=2;
DHDPS2_k_app_exp=1;
DHDPS2_Lys_Ki_app_exp=33;
DHDPS2_h_exp=2;
HSDHI_kforward_app_exp=0.84;
```

```
HSDHI_Thr_relative_residual_activity_app_exp=0.15;
HSDHI_Thr_relative_inhibition_app_exp=0.85;
HSDHI_Thr_Ki_app_exp=400;
HSDHII_kforward_app_exp=0.64;
HSDHII_Thr_relative_residual_activity_app_exp=0.25;
HSDHII_Thr_relative_inhibition_app_exp=0.75;
HSDHII_Thr_Ki_app_exp=8500;
HSK_kcat_app_exp=2.8;
HSK_Hser_app_exp=14;
TS1_kcatmin_exp=0.42;
TS1_AdoMet_kcatmax_exp=3.5;
TS1_AdoMEt_Km_no_AdoMet_exp=250;
TS1_AdoMet_Ka1_exp=73;
TS1_AdoMet_Ka2_exp=0.5;
TS1_AdoMet_Ka3_exp=1.09;
TS1_AdoMet_Ka4_exp=142;
TS1_Phosphate_Ki_exp=1000;
TS1_h_exp=2;
CGS_kcat_exp=30;
CGS_Cys_Km_exp=460;
CGS_Phser_Km_exp=2500;
CGS_Phosphate_Ki_exp=2000;
TD_k_app_exp=0.0124;
TD_Ile_Ki_no_Val_app_exp=30;
TD_Val_Ka1_app_exp=73;
TD_Val_Ka2_app_exp=615;
TD_h_app_exp=3;
Lys_tRNAS_Vmax=0.43;
Lys_tRNAS_Lys_Km=25;
Thr_tRNAS_Vmax=0.43;
Thr_tRNAS_Thr_Km=100;
Ile_tRNAS_Vmax=0.43;
Ile_tRNAS_Ile_Km=20;
LKR_kcat_exp=3.1;
LKR_Lys_Km_exp=13000;


% --------------------  Flux formulations --------------------------------%
Vak1 =AK1*(AK1_kforward_app_exp - AK1_kreverse_app_exp*y(1,:))./ ...
    (1+(y(3,:)/(AK1_Lys_Ki_app_exp/(1+AdoMet/
AK1_AdoMet_Ka_app_exp))).^AK1_h_exp)+s+z;
Vak2 = AK2*(AK2_kforward_app_exp - AK2_kreverse_app_exp*y(1,:))./ ...
    (1+(y(3,:)/ AK2_Lys_Ki_app_exp).^AK2_h_exp);
VakI =AKHSDHI*(AKI_kforward_app_exp - AKI_kreverse_app_exp*y(1,:)).*1./ ...
    (1+(y(6,:)/ AKI_Thr_Ki_app_exp).^AKI_h_exp);
VakII =AKHSDHII*(AKII_kforward_app_exp - AKII_kreverse_app_exp*y(1,:))./...
    (1+(y(6,:)/ AKII_Thr_Ki_app_exp).^AKII_h_exp);
Vasadh = ASADH*(ASADH_kforward_app_exp*y(1,:)-
ASADH_kreverse_app_exp*y(2,:))+s+z;
Vdhdps1 = DHDPS1* DHDPS1_k_app_exp
*y(2,:).*(1./(1+(y(3,:)/DHDPS1_Lys_Ki_app_exp).^DHDPS1_h_exp))+s;
Vdhdps2 = DHDPS2* DHDPS2_k_app_exp *y(2,:).*(1./(1+(y(3,:)/
DHDPS2_Lys_Ki_app_exp).^DHDPS2_h_exp));
VhsdhI = AKHSDHI* HSDHI_kforward_app_exp *y(2,:).*(
HSDHI_Thr_relative_residual_activity_app_exp + ...
    HSDHI_Thr_relative_inhibition_app_exp ./(1+y(6,:)/
HSDHI_Thr_Ki_app_exp))+z;
VhsdhII = AKHSDHII* HSDHII_kforward_app_exp *y(2,:).*(
HSDHII_Thr_relative_residual_activity_app_exp + ...
    HSDHII_Thr_relative_inhibition_app_exp ./(1+y(6,:)/
HSDHII_Thr_Ki_app_exp));
Vhsk = HSK* HSK_kcat_app_exp *y(4,:)./( HSK_Hser_app_exp +y(4,:))+z;
```

```
Vts1=TS1*(TS1_kcatmin_exp + TS1_AdoMet_kcatmax_exp *AdoMet^TS1_h_exp
/TS1_AdoMet_Ka1_exp)/ ...
    (1+AdoMet^TS1_h_exp /
TS1_AdoMet_Ka1_exp)*y(5,:)./((1+Phosphate/TS1_Phosphate_Ki_exp)* ...
    ( TS1_AdoMEt_Km_no_AdoMet_exp *(1+AdoMet/ TS1_AdoMet_Ka2_exp)/...
    (1+AdoMet/ TS1_AdoMet_Ka3_exp))./(1+AdoMet^TS1_h_exp /
TS1_AdoMet_Ka4_exp)+y(5,:))+z;
% Vcgs= CGS*( CGS_kcat_exp /(1+ CGS_Cys_Km_exp /Cys))*y(5,:)./...
%     (( CGS_Phser_Km_exp /(1+ CGS_Cys_Km_exp /Cys)).*(1+Phosphate/
CGS_Phosphate_Ki_exp)+ y(5,:));
Vtd = TD*TD_k_app_exp*y(6,:)./(1+(y(7,:)/( TD_Ile_Ki_no_Val_app_exp +
TD_Val_Ka1_app_exp *Val/...
    ( TD_Val_Ka2_app_exp +Val))).^ TD_h_app_exp)+z;
VLys_tRNAS = Lys_tRNAS_Vmax *y(3,:)./(Lys_tRNAS_Lys_Km+y(3,:))+s;
VThr_tRNAS = Thr_tRNAS_Vmax *y(6,:)./(Thr_tRNAS_Thr_Km+y(6,:));
VIle_tRNAS =Ile_tRNAS_Vmax *y(7,:)./(Ile_tRNAS_Ile_Km+y(7,:))+z;
Vlkr= LKR*LKR_kcat_exp*y(3,:)./(LKR_Lys_Km_exp+y(3,:));

% ------------------ fluxes are the main outputs of this function ------- %
fluxes = [Vak1 + Vak2 + VakI + VakII;Vasadh;VhsdhI+VhsdhII;Vdhdps1+Vdhdps2;...
    Vlkr+VLys_tRNAS;Vhsk;Vts1;VThr_tRNAS;Vtd;VIle_tRNAS];

% --- xdots are the secondary outputs of this function in case needed for
% trouble shooting --------------------------------------------------------- %
xdot = zeros(size(y));

% dydt(1)  = vAK - vASADH ;
xdot(1,:) = Vak1 + Vak2 + VakI + VakII - Vasadh;
% dydt(2)  = vASADH - vDHDPS - vHSDH ;
xdot(2,:) = Vasadh - Vdhdps1 -Vdhdps2- VhsdhI -VhsdhII ;
% dydt(3)  = vDHDPS - vLKR ;
xdot(3,:) = Vdhdps1 + Vdhdps2 - Vlkr - VLys_tRNAS;
% dydt(4)  = vHSDH - vHSK ;
xdot(4,:) = VhsdhI + VhsdhII -Vhsk;
% dydt(5)  = vHSK - vTS1 - Vcgs;
xdot(5,:) = Vhsk  - Vts1;
% dydt(6)  = vTS1 - vTD - vThr_tRNAsth;
xdot(6,:) = Vts1 - Vtd - VThr_tRNAS;
% dydt(7)  = Vtd-VIle_tRNAS;
xdot(7,:) = Vtd-VIle_tRNAS;
% dydt(8)  = vThr_tRNAsth;
xdot(8,:) = VThr_tRNAS;
end
```

Next, the MATLAB script *main_analysis.m* shows other steps of the analysis, which are outlined in comments throughout the MATLAB script and the main article.

main_analysis.m

```
function z=main_analysis(sd)

% ---------------------------------------------------------------------- %
%
% This is the main analysis function, which calls the ODE, fluxes and the
% rest of the functions and plots the results depicted in the main article.
%
% ---------------------------------------------------------------------- %
close all
clear all
%% ------------------ Run Diff_Curien ODEs ------------------------------ %
Y0 = zeros(8,1); %Initial condition
```

```
step = 1e-2;
t=0:step:1.5e3;
[T,X] = ode15s(@(t,y) Diff_Curien_sz(t,y,0,0),t,Y0);

figure(1)
plot(T,X,'linewidth',2.5)
legend('X_1','X_2','X_3','X_4','X_5','X_6','X_7','X_8')
xlabel('time [min]','FontSize',16)
```

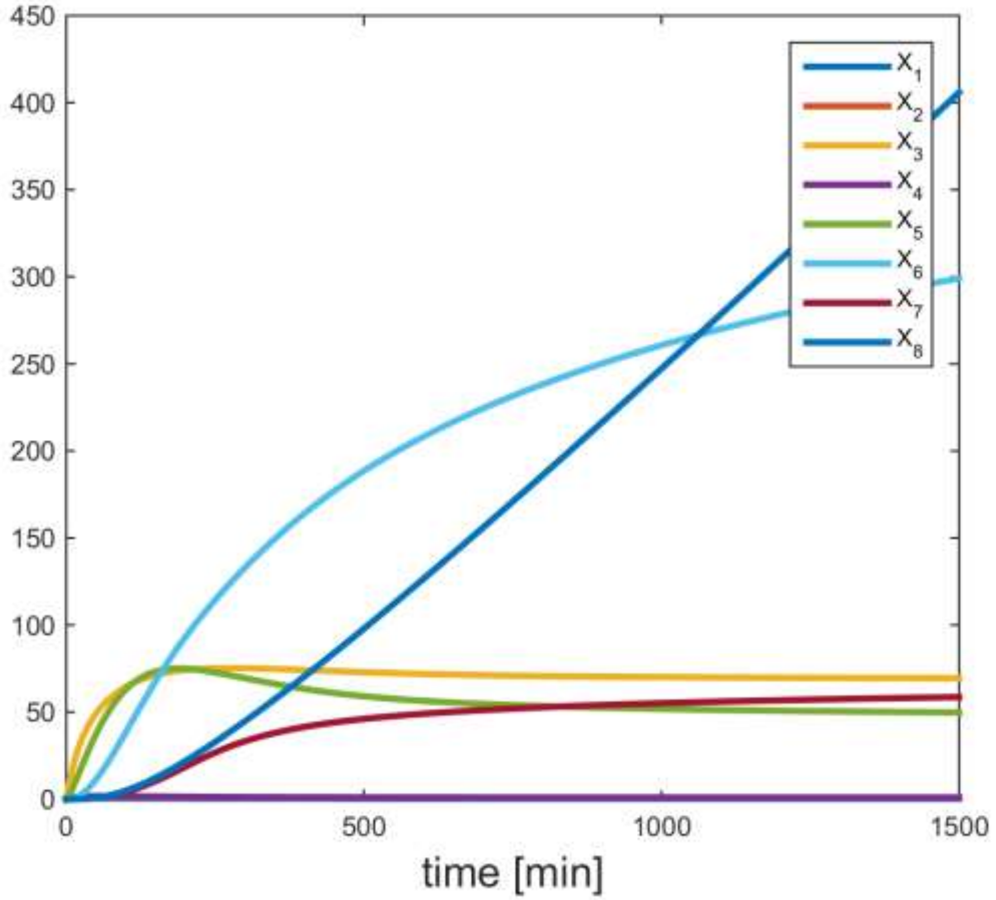

Figure 1- Metabolite concentrations plotted *vs.* time for the Curien model.

```
%% ----------- Run Fluxes_Curien to compare with analysis output---------- %
[fluxes,Xdot] = Fluxes_Curien(T,X');

figure(2)
plot(T,fluxes,'linewidth',2.5)
legend('v_1','v_2','v_3','v_4','v_5','v_6','v_7','v_8','v_9','v_{10}')
xlabel('time [min]','FontSize',16)
```

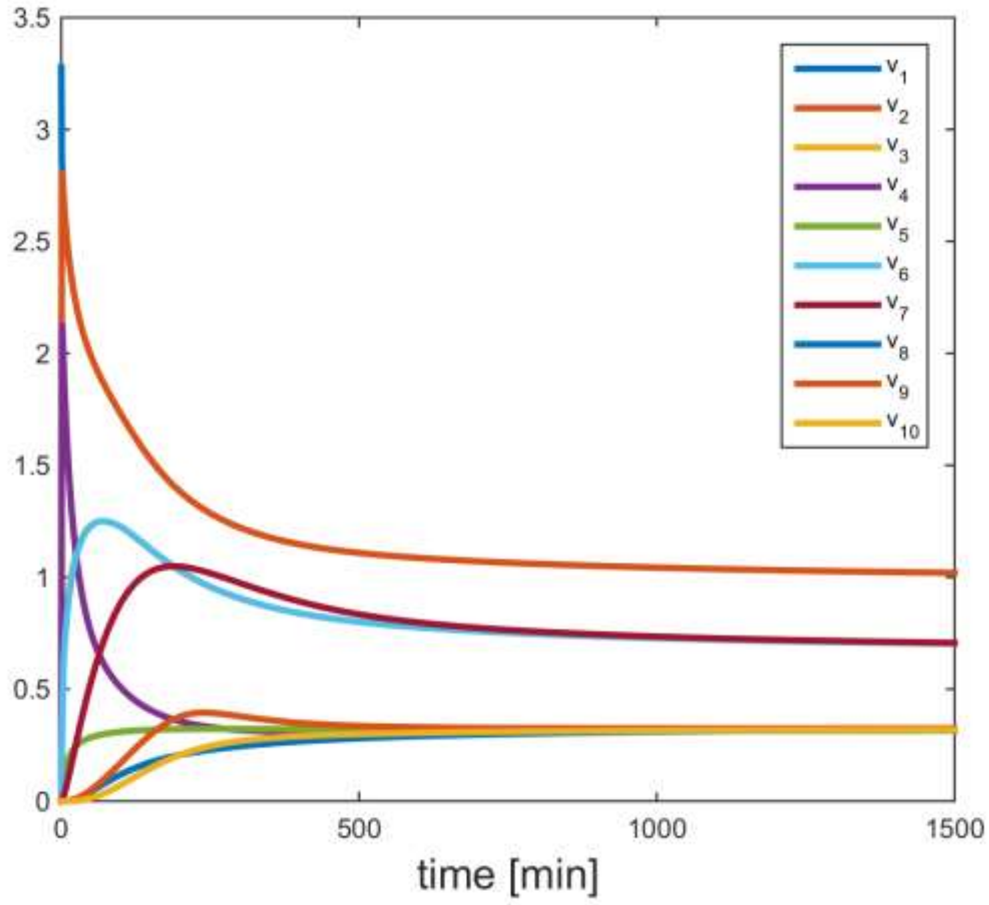

Figure 2- Original fluxes of the Curien model *vs.* time.

```
%% ------------ Translate the stoichiometry and pathway structure into the
% Stoi matrix below ----------------------------------------------------- %
Stoi = [1 , -1 ,  0 ,  0 ,  0 ,  0 ,  0  ,  0 ,  0 ,  0
        0 , 1  , -1 , -1 ,  0 ,  0 ,  0  ,  0 ,  0 ,  0
        0 , 0  ,  0 ,  1 , -1 ,  0 ,  0  ,  0 ,  0 ,  0
        0 , 0  ,  1 ,  0 ,  0 , -1 ,  0  ,  0 ,  0 ,  0
        0 , 0  ,  0 ,  0 ,  0 ,  1 , -1  ,  0 ,  0 ,  0
        0 , 0  ,  0 ,  0 ,  0 ,  0 ,  1  , -1 , -1 ,  0
        0 , 0  ,  0 ,  0 ,  0 ,  0 ,  0  ,  0 ,  1 , -1
        0 , 0  ,  0 ,  0 ,  0 ,  0 ,  0  ,  1 ,  0 ,  0];
A = Stoi;
save Curien_Xdot Xdot A
% ----------- NullSpace is a matrix with each columns constituting a basis
% for the null space; here these are two 10-dimensional vectors, i.e., we have
a 10 by 2 NullSpace ---------------------------------------------------- %
NullSpace = null(Stoi);

% NullSpace =
%
%     0.5374    0.0534
%     0.5374    0.0534
%     0.1162    0.3914
%     0.4212   -0.3380
%     0.4212   -0.3380
%     0.1162    0.3914
```

```
%      0.1162    0.3914
%     -0.0000    0.0000
%      0.1162    0.3914
%      0.1162    0.3914

% ----------- Gammas are simply the projection of the fluxes on the
% nullspace ------------------------------------------------------------ %
Gammas = NullSpace'*fluxes;
figure(3)
plot(Gammas(1,:),Gammas(2,:),'.')
arrow('start',Gammas(:,1:900:5000)','stop',Gammas(:,2:900:5000)')
 xlabel('\gamma_1','FontSize',16)
 ylabel('\gamma_2','FontSize',16)
```

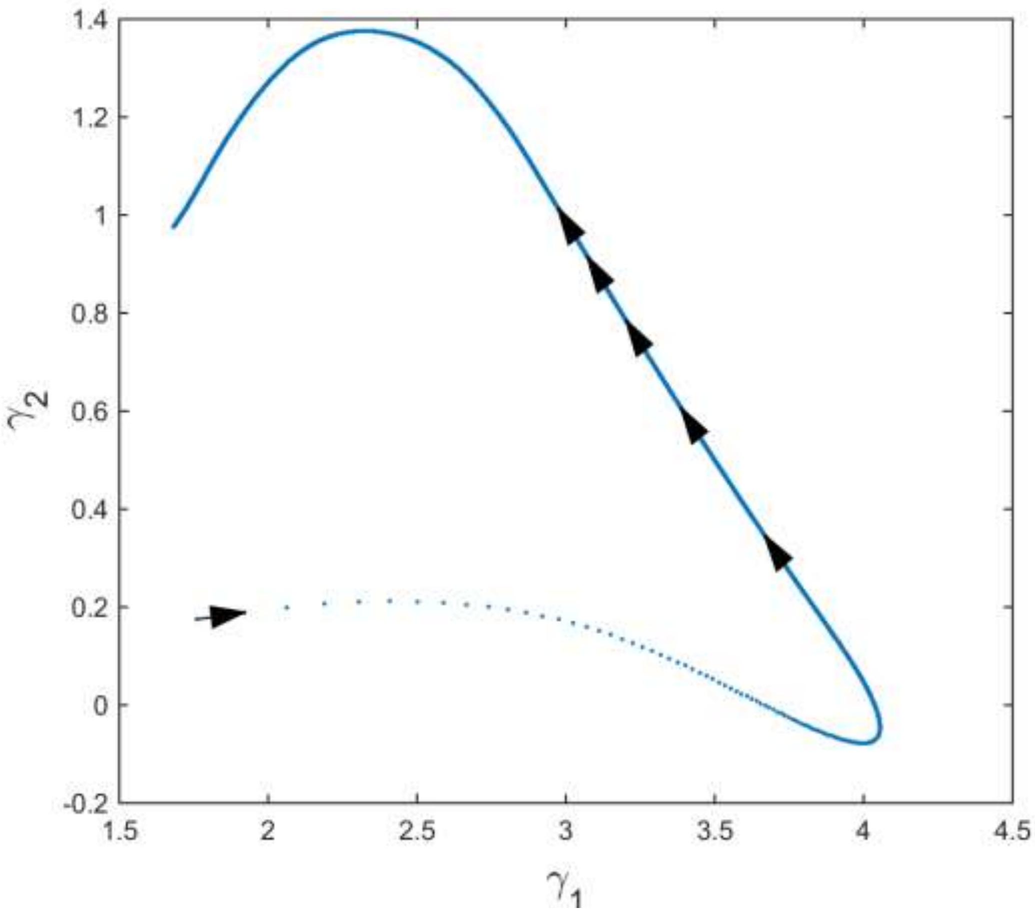

Figure 3- Gamma trajectory for the Curien model (Figure 5 of the main text).

```
%%
b = Xdot; % Each row is Xi_dot
figure(4)
plot(Gammas(1,:),Gammas(2,:),'.')
arrow('start',Gammas(:,1:900:5000)','stop',Gammas(:,2:900:5000)')
for j = 1:7
hold on
lb = [-1,-1];
ub = [10.5,Inf];
c = -pinv(A)*b(:,j);
% c = -A\b(:,j);
```

```
plotregion(NullSpace,c,lb,ub,'r',0.1)
end
axis equal
xlim([0.5,4.1])
ylim([-0.7,2])
xlabel('\gamma_1','FontSize',16)
ylabel('\gamma_2','FontSize',16)


%% ------- Finding corners of the non-negative fluxes in Gamma-space -------- %
% For numerical reasons only, and only for this section of finding the
% corners, we remove the effect of the fully determined v10 and redefine the
% vectors b consisting of time derivatives of all metabolites but X8 and
% the 7 by 9 Stoichiometric matrix A9.

% The numerical issues are caused because NullSpace has zero entries for v8
% but MATLAB calculation error causes the entries to be a very small on the
% order of 1e-15 but non-zero entries, which introduces an unwanted new
% inequality constraint.
A9 = [1 , -1 , 0 , 0 , 0 , 0 , 0  , 0 , 0
      0 , 1  ,-1 ,-1 , 0 , 0 , 0  , 0 , 0
      0 , 0  , 0 , 1 ,-1 , 0 , 0  , 0 , 0
      0 , 0  , 1 , 0 , 0 ,-1 , 0  , 0 , 0
      0 , 0  , 0 , 0 , 0 , 1 ,-1  , 0 , 0
      0 , 0  , 0 , 0 , 0 , 0 , 1  ,-1 , 0
      0 , 0  , 0 , 0 , 0 , 0 , 0  , 1 , -1 ];

NullSpace9 = null(A9);%(9x2)
v8 = fluxes(8,:);
a = zeros(size(Xdot)); a(6,:) = v8;
b9 = Xdot(1:7,:) + a(1:7,:);
fluxes9 = fluxes([1:7,9:10],:);
Corners_init = zeros(size(b9,2),2);
j=0;
%%
for k=1:size(b9,2)
    j=j+1;
    cc = [pinv(A9)*b9(:,k);10000];
    AA = [-NullSpace9;1 0];
    [VV,nr,nre]=lcon2vert(AA,cc,[],[]);
    Corners_init(j,:) = VV(1,:);
end

figure(4)
plot(Corners_init(:,1),Corners_init(:,2),'.k')
xlabel('\gamma_1','FontSize',16)
ylabel('\gamma_2','FontSize',16)
```

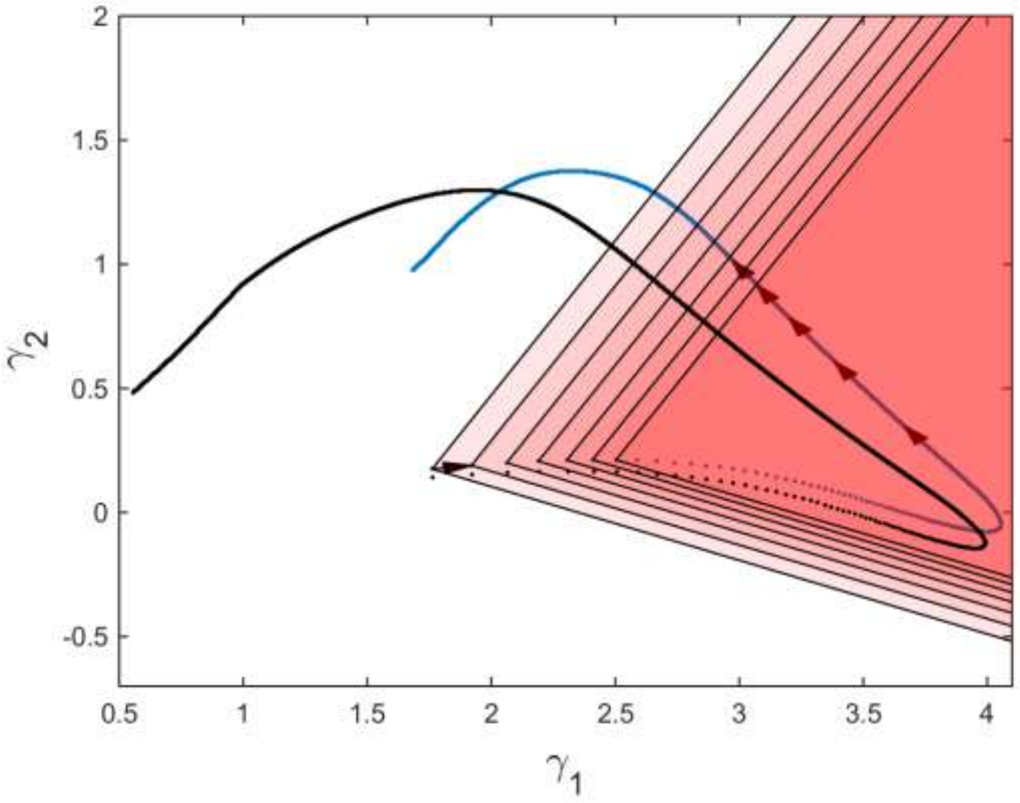

Figure 4- This figure corresponds to Figure 7B of the main text.

```
%% Adding a constant amount to the fluxes in Sets 1 and 2 of fluxes %%
% Here, two parameters s and z are introduced to show
% that addition of an equal amount (=s) to certain fluxes belonging to Set 1
% and/or an equal amount (=z) to Set 2 does not change the resulting
% metabolite concentrations. Figure 5 shows how Figure 7A of the main
% article is plotted.
s_list=[0,2,4,6,8];
z_list=[0,2,4,6,8];
l=0;
Gam = zeros(2,5,5);
figure(5)
for i=s_list(1:5)
    l=l+1;
    k=0;
    for j=z_list(1:5)
        k=k+1;
        [T,X] = ode15s(@(t,y) Diff_Curien_sz(t,y,i,j),t,Y0);
        fluxes = Fluxes_Curien_sz(T,X',i,j);
        Gammas = NullSpace'*fluxes;
        Gam(:,l,k)= Gammas(:,2500);
        hold on
        ax = gca;
        ax.ColorOrderIndex = 1;
        plot(Gammas(1,:),Gammas(2,:),'.')
```

```
    end
end
Gam1 = reshape(Gam(:,1,:),[2,5]);
Gam2 = reshape(Gam(:,:,1),[2,5]);
hold on
plot(Gam1(1,:),Gam1(2,:),'-oc','linewidth',2)
plot(Gam2(1,:),Gam2(2,:),'-or','linewidth',2)
 xlabel('\gamma_1','FontSize',16)
 ylabel('\gamma_2','FontSize',16)
 hold off
 end
```

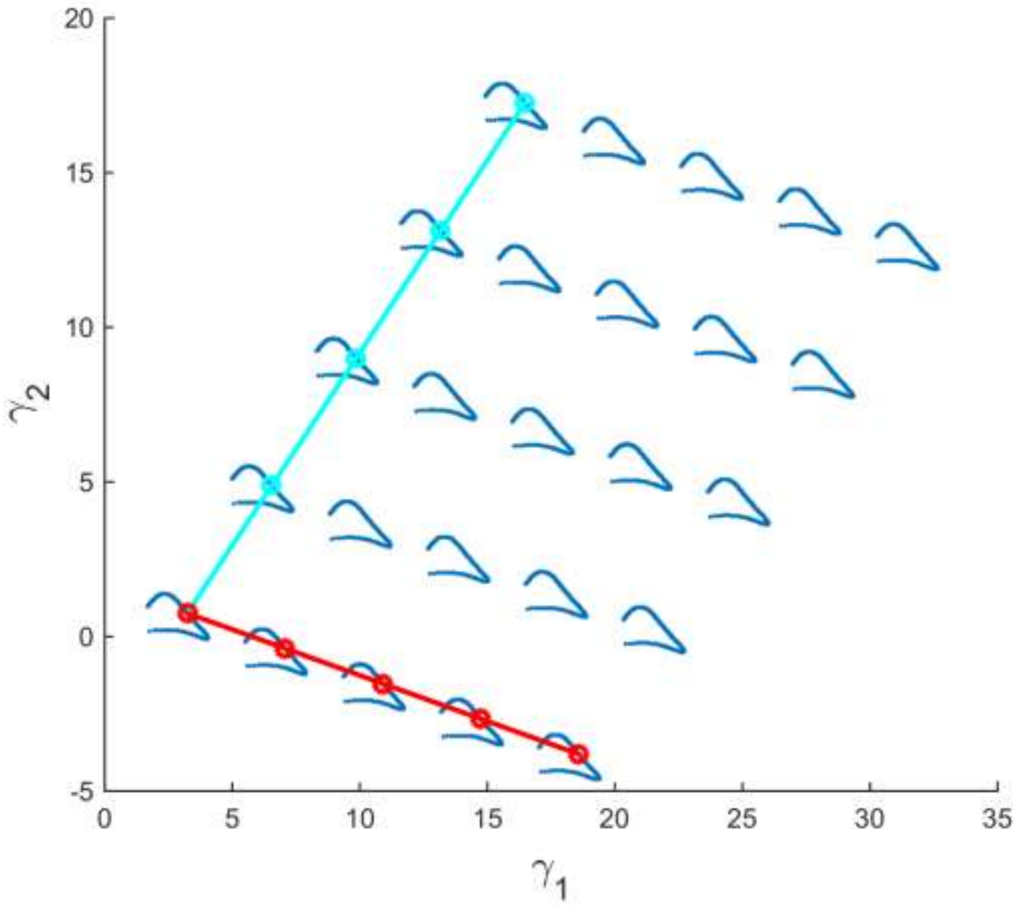

Figure 5- This figure is the same as Figure 7A of the main text.

Some necessary files in the MATLAB working directory are the following files taken from MATLAB file exchange website. These were mostly used for plotting the Gamma subspace corresponding to non-negative fluxes and are listed below:
arrow.m, written by Eric Johnson was used to plot the Gamma trajectory of Figure 7B. If using MATLAB 2014b or newer, the code needed to be slightly modified. The modification was done according to instructions in the comment section of the corresponding page on MATLAB file exchange website and are provided in the github folder for convenience.

plotregion.m written by Per Bergström plots convex closed regions in 2D/3D. The region is a subset of R^2 or R^3 such that $Ax \geq b$ and $lb \leq x \leq ub$. It is also possible to plot points in the same plot.
lcon2vert.m and vert2lcon written by Matt J is used for finding the vertices of a bound polyhedron in R^n. These files are a part of a group of codes for analyzing polyhedra presented in the form of linear inequalities. Addition of an upper bound was needed to make the Gamma subspace corresponding to the non-negative fluxes a bounded polyhedron in order to be able to use these functions.

Please note that Figures 3 and 4, plotted in the *analysis.m* MATLAB script, correspond to Figures 5 and 7B in the main article.

2. Feasible non-negative solutions can manifest diverse dynamics

Figures 3C and 6 show feasible stable non-negative fluxes constructed using an LTI state space representation $\dot{\gamma} = B\gamma$. For this construction, we constrained the matrix $B = \begin{pmatrix} b_{11} & b_{12} \\ b_{12} & b_{22} \end{pmatrix}$ such that the system converges toward a stable steady state similar to the biological system under study. We are interested in stable systems for which $det(B) > 0$ and $trace(B) \leq 0$. In the following MATLAB script, we choose b_{11}, b_{12}, and b_{13} randomly from the interval $[-1,1]$, and b_{22} from $[-1 - b_{11}, -b_{11}]$. This way $trace(B) = b_{11} + b_{22} \leq 0$. We further select for the matrices with a positive determinant. These two constraints eliminate the possibility of B having negative eigenvalues and unstable trajectories.

The following MATLAB code was used to produce some feasible solutions (*MonteCarlo_nonnegatve_Curien.m*). We would like to emphasize that these solutions are a small subset of all feasible solutions, *i.e.* LTI systems with constrained B elements. Also we showed previously that moving the trajectory in the gamma-space does not change the metabolite concentrations, so here we shift trajectories to start at a certain $[\gamma_1(0), \gamma_2(0)]$ in order to get more trajectories resulting in non-negative fluxes faster. Please note that the purpose of this section is to show that the underlying dynamic behaviors of non-negative fluxes can result in the same measured metabolite concentrations. Later on we will plot (*Plot_nonnegative_MonteCarlo.m*) these fluxes against the shifted version of the original fluxes only for better visual comparison of the dynamic behaviors.

MonteCarlo_nonnegative_Curien.m

```
% For plotting purposes only, the trajectories are shifted such that they all
start from Gamma(0)
Y0=[16,3];

% This will be the matrix containing the i-th B resulting in
% non-negative fluxes in the form of the vector [b11,b12,b21,b22]^T as its
```

```
% i-th column.
Y1 = []; % This will contain the corresponding Gamma1 as its i-th column
Y2 = []; % This will contain the corresponding Gamma2 as its i-th column
VV = []; % And finally the corresponding fluxes vs. time are stored in VV.
% If this script finds m set of non-negative fluxes, then BB is 4 by m, Y1
% and Y2 are l by m, and VV is an l by m*10

load Curien_data % This has Xdot (7x1501) for T=0:0.1:150 stored in it, as
% well as matrix A (the 7x9 version)
% It also contains Curien fluxes v1,...,v7,v9,v10, which are not used in
% this script.

step = 1e-1;
t=0:step:1.5e2;
NullSpace = null(A);

for i = 1:1e5
    b11 = -1+2*rand; % in [-1,1]
    b22 = -b11-rand; % in [1-b11,b11]
    b12 = -1+2*rand; % in [-1,1]
    b21 = -1+2*rand; % in [-1,1]
    % This choice of b22 results in trace(B) = b11+b22 <=0
    deter=b11*b22-b12*b21;

    if deter>0
        B=[b11 b12;b21 b22];
        [T,Y] = ode45(@(t,y) Diff_gamma(t,y,B),t,Y0);
        V = pinv(A)*Xdot + NullSpace*Y';
        if min(min(V))>= -1e-4
            disp('Found one')
            Y1 =[Y1,Y(:,1)];
            Y2 =[Y2,Y(:,2)];
            BB=[BB,reshape(B,4,1)];
            VV=[VV;V];
        end
    end
end
save NonNegative_V Y1 Y2 BB VV T
```

Plot_nonnegative_MonteCarlo.m

```
load NonNegative_V % Contains Y1 Y2 BB VV : 2082 working trajectories in
% Gamma plane that result in non-negative fluxes
% BB (4x2082) , VV(18738x5001) , Y1,Y2 (5001x2082)

load Curien_data_150 % Contains Curien fluxes v1,...,v7,v9,v10 and the
% corresponding T = 0:0.1:150
% It also has Xdot (7x1501) for t=0:0.1:150 stored in it, as
% well as matrix A (the 7x9 version)
ind=[1:7,9:10]; %v8 is not included since it's fully determined.

% ------------------------------ A number of flux sets that are relatively
% visually diverse are selected for plotting ---------------------------- %
smallsetind = [71,619,1417,49,12,45,11,2];

figure(6)
for i=1:9
subplot(3,3,i)
plot(T,VV(i:9:end,:))
hold on
plot(T,fluxes(i,:)+VV(i,1)-fluxes(i,1),'.k')
xlim([0,150])
```

```
str = sprintf('v_{%d}',ind(i));
if (i>6)&&(i<10)
xlabel('time [$s$]','Interpreter','latex')
end
if (i==1)|(i==4)|(i==7)
ylabel('Flux [$\mu M^{-1}.s^{-1}$]','Interpreter','latex')
end
title(str);
hold off
end
```

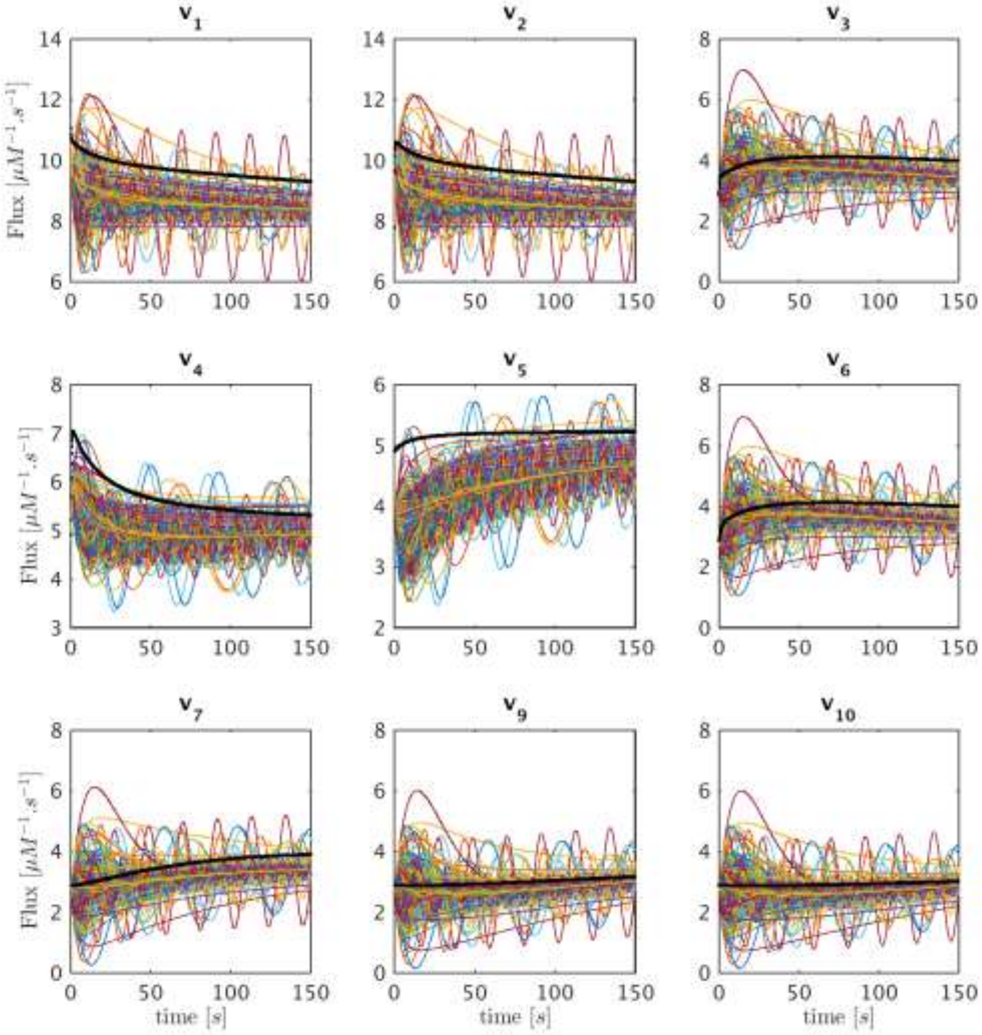

Figure 6- This figure is similar to Figure 6 of the main text, except that here a larger set of nonnegative fluxes is plotted.

3. Minimum-norm solutions

The goal of the following MATLAB script *Optim_Curien_fluxes.m* is to calculate the minimum norm flux sets, and confirming that minimizing L_1 and L_2–norms result in

the exact same set of fluxes and corresponding Gamma trajectories. The first section of the code solves an optimization problem using quadratic programing for every single point in time, and might take a while depending on the computer specifications. For a quick comparison, the linear programing problem is solved only for 1 out of every 100 points in Section 2 of the script.
This script is solving the minimum norm flux in *min_norm_solution*.

Optim_Curien_fluxes.m

```
load Curien_data % Contains T fluxes Xdot X Gammas A
load Corners_init % Contains Corners_init
%% ------------ Quadratic programing: minimizing L2 norm --------------- %%
v_quad = zeros(10,size(Xdot,2));
H=eye(10);
f=zeros(10,1);
for i=1:size(Xdot,2)
v_quad(:,i) = quadprog(H,f,[],[],A,Xdot(:,i),zeros(10,1),[]);
end
Gammas_quad = NullSpace'*v_quad;
save min_norm_solution v_quad Gammas_quad T

%% --Linear prog. only for a subset of time points -> faster--%%

v_lin = zeros(10,size(Xdot,2));
f=ones(10,1);
for i=1:100:size(Xdot,2)
v_lin(:,i) = linprog(f,[],[],A,Xdot(:,i),zeros(10,1),[]);
end
Gammas_lin = NullSpace'*v_lin(:,1:100:size(Xdot,2));

%% ------------- Plotting the Gamma trajectories ------------ %%
figure(7)
hold on
plot(Gammas(1,:),Gammas(2,:),'.')
plot(Gammas_quad(1,:),Gammas_quad(2,:),'.')
plot(Gammas_lin(1,:),Gammas_lin(2,:),'.')
plot(Corners_init(:,1),Corners_init(:,2),'.k')

legend('Actual Gamma trajectory','Lin. prog','Quad. prog','Cone corners')
xlabel('\gamma_1','FontSize',16)
ylabel('\gamma_2','FontSize',16)
hold off
```

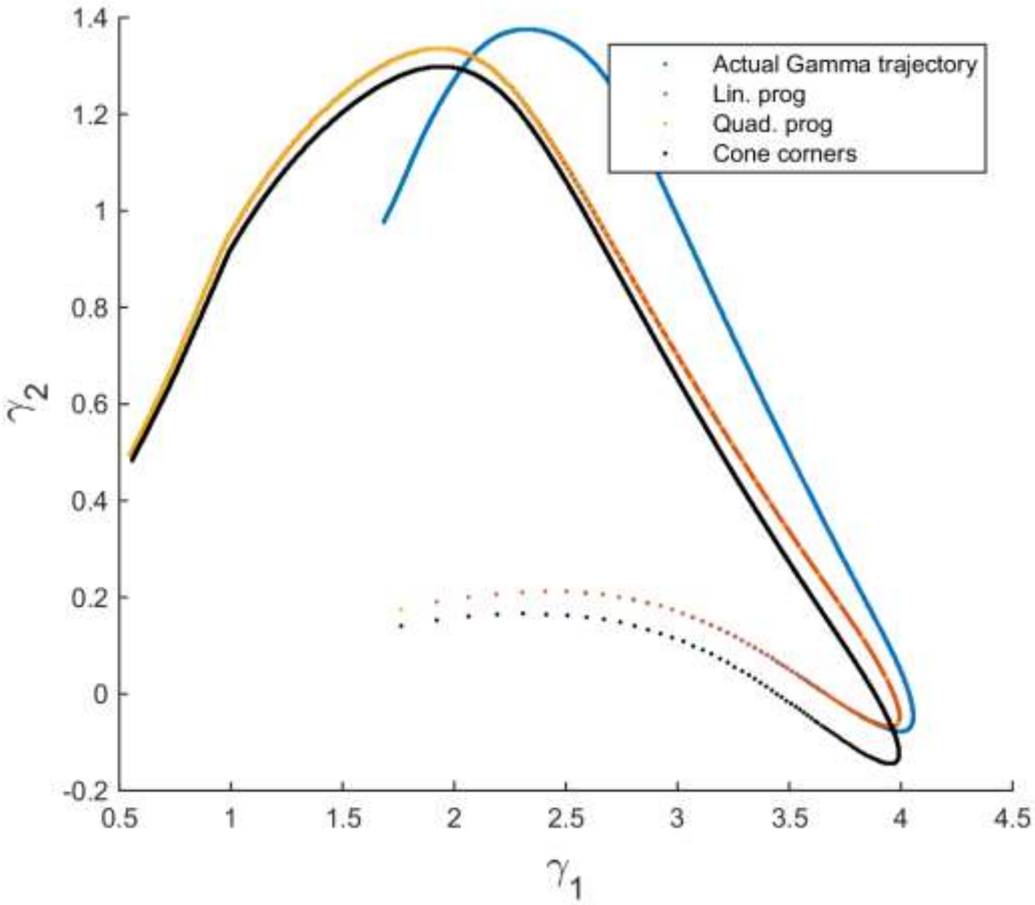

Figure 7- This figure shows (1) in blue, the actual Gamma trajectory of the Curien model, (2) in red, the Gamma trajectory corresponding to the minimum L1-norm flux set calculated by solving a constrained linear programing problem and almost completely covered by (3) the yellow line, depicting the Gamma trajectory corresponding to the minimum L2-norm sum of fluxes calculated by solving a constrained quadratic programing problem. Finally, (4) the corners of the feasible cones calculated previously and plotted in Figure S4 (Figure7B of the main text) are plotted in black. In theory this should be the same as the Lin. Prog and Quad. Prog results and the slight difference is due to the numerical error in the calculation of the corners.

4. Plotting the minimum-energy flux set and incorporating other relevant constraints (Figures 8-11)

Plot_constrained_solutions.m

```
load min_norm_solution % Contains v_quad, Gammas_quad, and T
load Curien_data % Contains Curien fluxes, Xdot, X, and Gammas as well aso
% the corresponding T = 0:0.01:1500, and the matrix A (the 8x10 version)

index = [1:7,9:10]; %v8 is not included since it's fully determined.
v_minnorm = v_quad(index,:);
V = fluxes(index,:);

%% -----------------------------------------------------------------%
```

```matlab
% -------------------- Plotting fluxes vs. time ---------------------%
% -----------------------------------------------------------------%
figure(8)
for i=1:9
subplot(3,3,i)
plot(T,V(i,:))
hold on
plot(T,v_minnorm(i,:))

str = sprintf('v_{%d}',ind(i));
if (i>6)&&(i<10)
    xlabel('time [$s$]','Interpreter','latex')
end
if (i==1)|(i==4)|(i==7)
    ylabel('Flux [$\mu M^{-1}.s^{-1}$]','Interpreter','latex')
end
if (i==1)
    legend('Actual','Min-energy')
end
title(str);
hold off
xlim([0 1500])
end
```

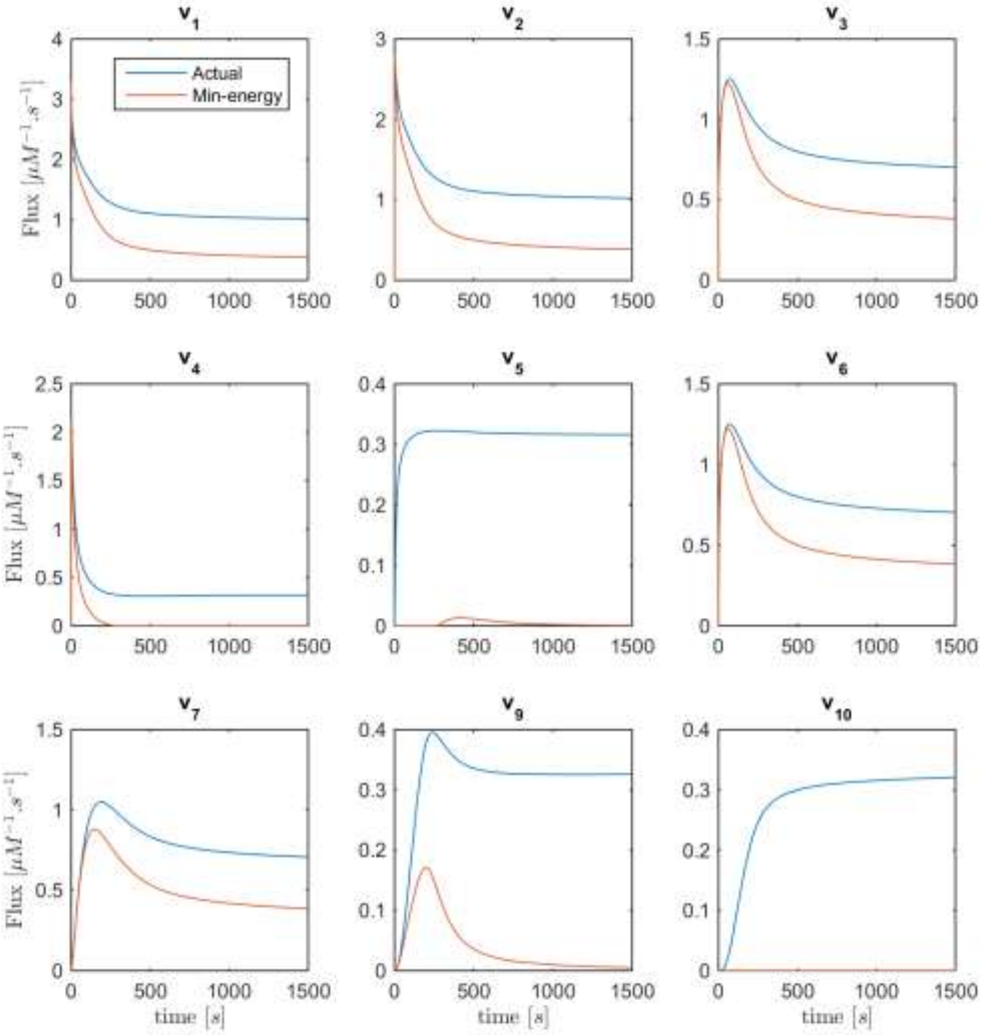

Figure 8- (Same as Figure 8) Fluxes v_1 to v_{10} with the exception of v_8 are plotted *vs.* time. Curves in red are the min-energy fluxes, while the blue curves show the actual fluxes of the Curien model. Flux v_8 is not shown because it belongs to the full-rank subset of the system and can be recovered exactly.

```
%% ------------------------------------------------------------------%
% ---------------- Plotting fluxes vs. their substrates -------------%
% ------------------------------------------------------------------%
% ---------------- One-substrate fluxes ----------------------------%
figure(9)
subplot(4,1,1)
title('$v_5$ $vs.$ $X_3$','Interpreter','latex')
hold on
plot(X(:,3),v_minnorm(5,:))
hold on
plot(X(:,3),V(5,:))
hold off
subplot(4,1,2)
hold on
title('$v_6$ $vs.$ $X_4$','Interpreter','latex')
plot(X(:,4),v_minnorm(6,:))
```

```
hold on
plot(X(:,4),V(6,:))
hold off
subplot(4,1,3)
hold on
title('$v_7$ $vs.$ $X_5$','Interpreter','latex')
plot(X(:,5),v_minnorm(7,:))
hold on
plot(X(:,5),V(7,:))
hold off
subplot(4,1,4)
hold on
title('$v_{10}$ $vs.$ $X_7$','Interpreter','latex')
plot(X(:,7),v_minnorm(9,:))
hold on
plot(X(:,7),V(9,:))
hold off
```

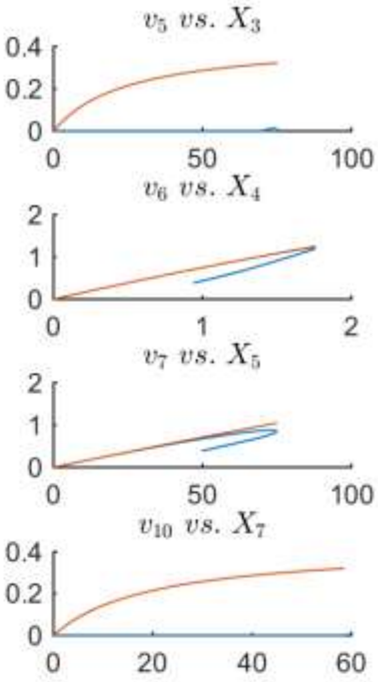

Figure 9- One-substrate fluxes *vs.* their substrates.

```
%% ------------------ Two-substrate fluxes --------------------------- %
figure(10)
subplot(4,1,1)
title('$v_5$ $vs.$ $X_3$','Interpreter','latex')
hold on
plot(X(:,3),v_minnorm_new(5,:))
hold on
plot(X(:,3),V(5,:))
hold off
subplot(4,1,2)
hold on
title('$v_6$ $vs.$ $X_4$','Interpreter','latex')
plot(X(:,4),v_minnorm_new(6,:))
hold on
plot(X(:,4),V(6,:))
```

```
hold off
subplot(4,1,3)
hold on
title('$v_7$ $vs.$ $X_5$','Interpreter','latex')
plot(X(:,5),v_minnorm_new(7,:))
hold on
plot(X(:,5),V(7,:))
hold off
subplot(4,1,4)
hold on
title('$v_{10}$ $vs.$ $X_7$','Interpreter','latex')
plot(X(:,7),v_minnorm_new(9,:))
hold on
plot(X(:,7),V(9,:))
hold off
```

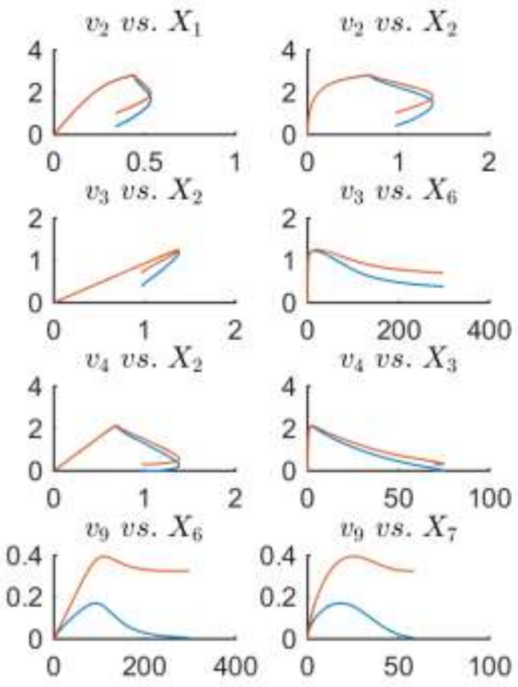

Figure 10

```
%% ---------------- Three-substrate flux v1 ------------------------- %
figure(11)
subplot(1,3,1)
title('$v_1$ $vs.$ $X_1$','Interpreter','latex')
hold on
plot(X(:,1),v_minnorm(1,:))
hold on
plot(X(:,1),V(1,:))
hold off
subplot(1,3,2)
title('$v_1$ $vs.$ $X_3$','Interpreter','latex')
hold on
plot(X(:,3),v_minnorm(1,:))
hold on
plot(X(:,3),V(1,:))
hold off
subplot(1,3,3)
title('$v_1$ $vs.$ $X_6$','Interpreter','latex')
```

```
hold on
plot(X(:,6),v_minnorm(1,:))
hold on
plot(X(:,6),V(1,:))
hold off
```

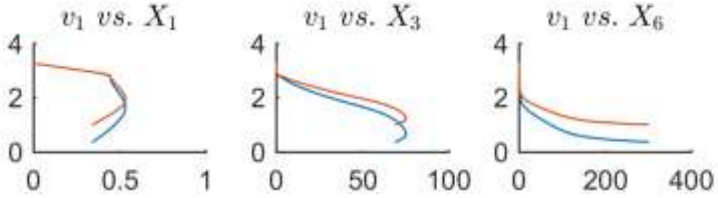

Figure 11

```
%% --------------------------------------------------------------------%
% ---------- Removing the folding-over phenomenon for v6  ---------- %
% --------------------------------------------------------------------%
[Y,Ind] = max(v_minnorm(6,:));
X4 = X(:,4);
v6 = v_minnorm(6,1:Ind);
v6q = interp1(X4(1:Ind)',v6,X4(Ind+1:end),'linear','extrap');

figure(12)
subplot(1,2,1)
plot(X4(1:Ind)',v6,'.')
hold on
plot(X4(Ind+1:end)',v_minnorm(6,Ind+1:end),'.r')
hold on
plot(X4(Ind+1:end),v6q,'.g')
legend('v_6','folded-over section of v_6','mapped to initial
branch','location','northwest')
xlabel('$X_4$ [$\mu M^{-1}$]','Interpreter','latex')
ylabel('$v_6$ flux [$\mu M^{-1}.s^{-1}$]','Interpreter','latex')
hold  off

subplot(1,2,2)
plot(T,V(6,:),'.r')
hold on
plot(T,[v6,v6q'],'.')
legend('v_6',' v_{6.new}')
xlabel('time [$s$]','Interpreter','latex')
ylabel('$v_6$ flux [$\mu M^{-1}.s^{-1}$]','Interpreter','latex')
hold off
```

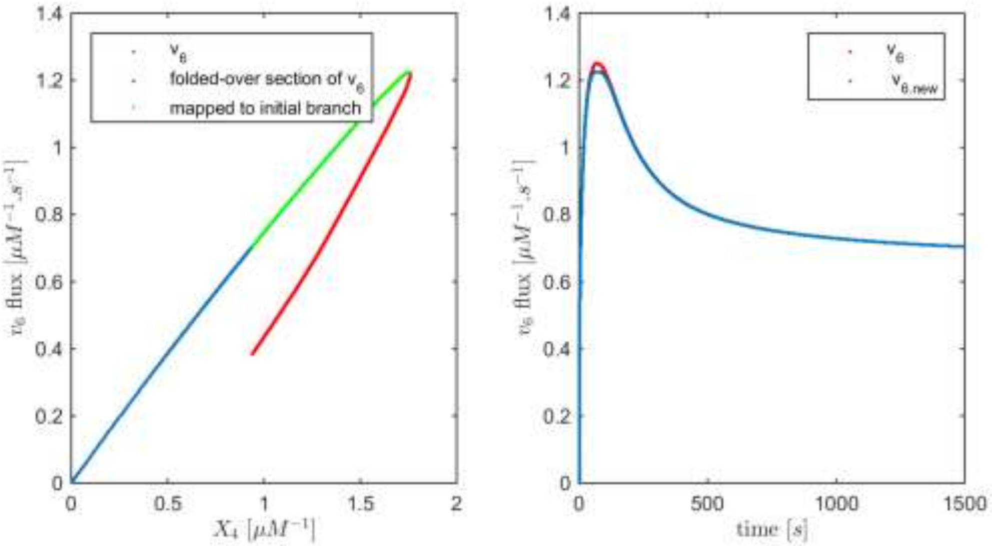

Figure 12- Left panel shows a close-up of flux v_6 *vs.* its only substrate X_4 in blue. The red folded-over segment was removed and v_6 for the time points where v_6 was on that folded-over branch were replaced by the values from the top branch for the corresponding X_4. The panel on the right shows that v_6 is almost accurately determined.

```
v6_new = [v6,v6q'];

Xdot_new = Xdot+[0;0;0;1;-1;0;0;0]*v6_new;
A_new = [A(:,1:5),A(:,7:10)];
V_new = [V(1:5,:);V(7:9,:)];
size(Xdot_new)
size(A_new)
size(V_new)

%% -------------------------------------------------------------------%%
% -----------------  Re-calculating v_minnorm ------------------------ %
% ------------------------------------------------------------------   %
v_quad_new = zeros(9,size(Xdot_new,2));
H=eye(9);
f=zeros(9,1);
for i=1:size(Xdot_new,2)
v_quad_new(:,i) = quadprog(H,f,[],[],A_new,Xdot_new(:,i),-1e-7*ones(9,1),[]);
%-1e-7 is set to overcome
end
Gammas_quad_new = NullSpace'*v_quad_new;

%% -----------------------------------------------------------------%
% -------------- The corrected fluxes vs. time --------------------- %
% ------------------------------------------------------------------%
v_temp = [v_quad_new(1:5,:);v6_new;v_quad_new(6:9,:)];
v_minnorm_new = v_temp(index,:);

figure(13)
for i=1:9
subplot(3,3,i)
plot(T,V(i,:))
hold on
plot(T,v_minnorm_new(i,:))

str = sprintf('v_{%d}',ind(i));
if (i>6)&&(i<10)
    xlabel('time [$s$]','Interpreter','latex')
```

```
end
if (i==1)|(i==4)|(i==7)
    ylabel('Flux [$\mu M^{-1}.s^{-1}$]','Interpreter','latex')
end
if (i==1)
    legend('Actual','Min-energy')
end
title(str);
hold off
xlim([0 1500])
end
```

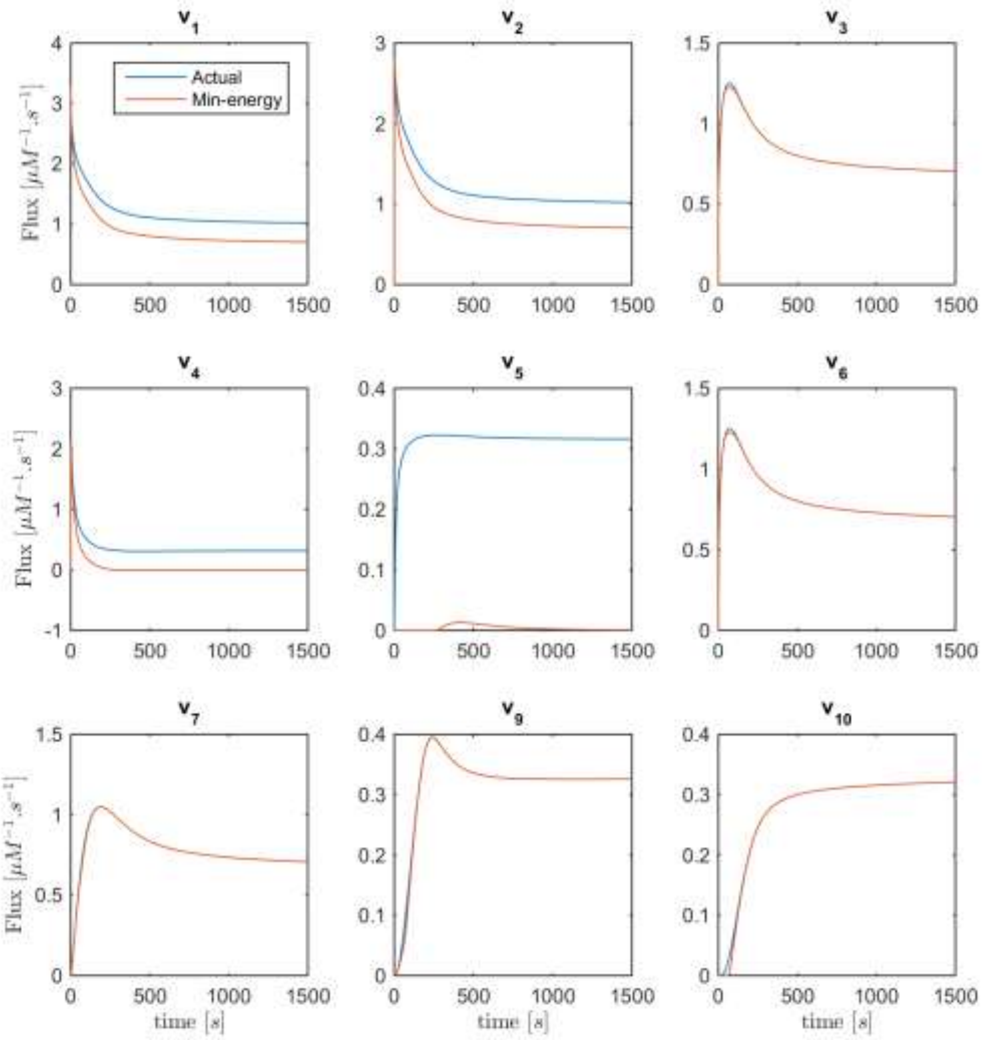

Figure 13

```
%% ------------------------------------------------------------------%
% ---------- Plotting corrected fluxes vs. their substrates ---------%
% ------------------------------------------------------------------%
% ---------------- One-substrate fluxes ----------------------------%
figure(14)
subplot(4,1,1)
```

```
title('$v_5$ $vs.$ $X_3$','Interpreter','latex')
hold on
plot(X(:,3),v_minnorm_new(5,:))
hold on
plot(X(:,3),V(5,:))
hold off
subplot(4,1,2)
hold on
title('$v_6$ $vs.$ $X_4$','Interpreter','latex')
plot(X(:,4),v_minnorm_new(6,:))
hold on
plot(X(:,4),V(6,:))
hold off
subplot(4,1,3)
hold on
title('$v_7$ $vs.$ $X_5$','Interpreter','latex')
plot(X(:,5),v_minnorm_new(7,:))
hold on
plot(X(:,5),V(7,:))
hold off
subplot(4,1,4)
hold on
title('$v_{10}$ $vs.$ $X_7$','Interpreter','latex')
plot(X(:,7),v_minnorm_new(9,:))
hold on
plot(X(:,7),V(9,:))
hold off
```

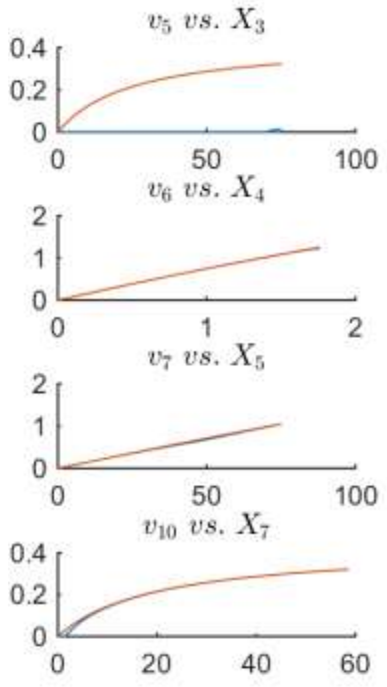

Figure 14

```
%% ------------------ Two-substrate fluxes --------------------------- %
figure(15)
subplot(4,2,1)
title('$v_2$ $vs.$ $X_1$','Interpreter','latex')
hold on
plot(X(:,1),v_minnorm_new(2,:))
```

```
hold on
plot(X(:,1),V(2,:))
hold off
subplot(4,2,2)
title('$v_2$ $vs.$ $X_2$','Interpreter','latex')
hold on
plot(X(:,2),v_minnorm_new(2,:))
hold on
plot(X(:,2),V(2,:))
hold off
subplot(4,2,3)
title('$v_3$ $vs.$ $X_2$','Interpreter','latex')
hold on
plot(X(:,2),v_minnorm_new(3,:))
hold on
plot(X(:,2),V(3,:))
hold off
subplot(4,2,4)
title('$v_3$ $vs.$ $X_6$','Interpreter','latex')
hold on
plot(X(:,6),v_minnorm_new(3,:))
hold on
plot(X(:,6),V(3,:))
hold off
subplot(4,2,5)
title('$v_4$ $vs.$ $X_2$','Interpreter','latex')
hold on
plot(X(:,2),v_minnorm_new(4,:))
hold on
plot(X(:,2),V(4,:))
hold off
subplot(4,2,6)
title('$v_4$ $vs.$ $X_3$','Interpreter','latex')
hold on
plot(X(:,3),v_minnorm_new(4,:))
hold on
plot(X(:,3),V(4,:))
hold off
subplot(4,2,7)
title('$v_9$ $vs.$ $X_6$','Interpreter','latex')
hold on
plot(X(:,6),v_minnorm_new(8,:))
hold on
plot(X(:,6),V(8,:))
hold off
subplot(4,2,8)
title('$v_9$ $vs.$ $X_7$','Interpreter','latex')
hold on
plot(X(:,7),v_minnorm_new(8,:))
hold on
plot(X(:,7),V(8,:))
hold off
```

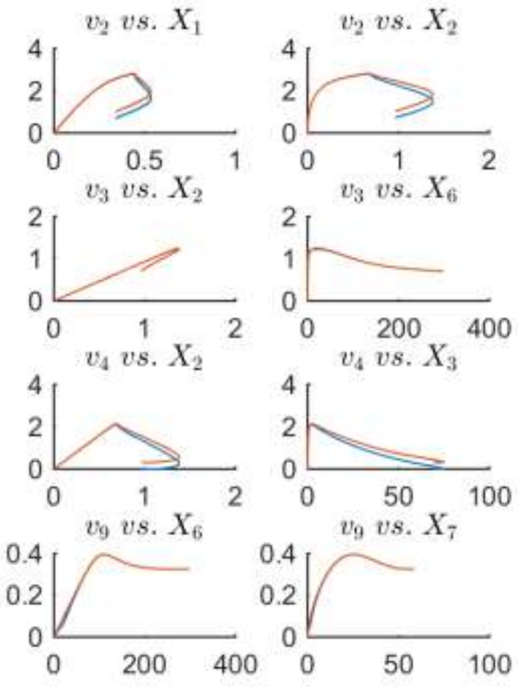

Figure 15

```
%% --------------- Three-substrate fluxes --------------------------- %
figure(16)
subplot(1,3,1)
title('$v_1$ $vs.$ $X_1$','Interpreter','latex')
hold on
plot(X(:,1),v_minnorm_new(1,:))
hold on
plot(X(:,1),V(1,:))
hold off
subplot(1,3,2)
title('$v_1$ $vs.$ $X_3$','Interpreter','latex')
hold on
plot(X(:,3),v_minnorm_new(1,:))
hold on
plot(X(:,3),V(1,:))
hold off
subplot(1,3,3)
title('$v_1$ $vs.$ $X_6$','Interpreter','latex')
hold on
plot(X(:,6),v_minnorm_new(1,:))
hold on
plot(X(:,6),V(1,:))
hold off
```

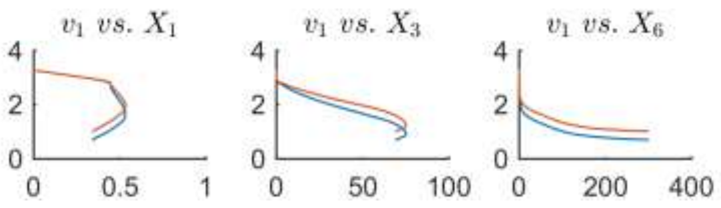

Figure 16

Supplemental references:

[1] https://github.com/sepidd/Identification-of-Metabolic-Pathway-Systems
[2] Johnson, Eric. “arrom.m” *MATLAB central file exchange*, 14 Jan 2000. Web. Dec 7 2015. <http://www.mathworks.com/matlabcentral/fileexchange/278-arrow-m>
[3] Bergström, Per. “plotregion.m” MATLAB central file exchange, 5 Dec 2005. Web. Dec 7 2015 <http://www.mathworks.com/matlabcentral/fileexchange/9261-plot-2d-3d-region>
[4] J, Matt. “Analyze N-dimensional Polyhedra in terms of Vertices or (In)Equalities” MATLAB central file exchange, 30 Mar 2011. Web. Dec 7 2015 <http://www.mathworks.com/matlabcentral/fileexchange/30892-analyze-n-dimensional-polyhedra-in-terms-of-vertices-or--in-equalities/content/lcon2vert.m>
